# α-Synuclein Amyloids Hijack Prion Protein to Gain Cell Entry, Facilitate Cell-to-Cell Spreading and Block Prion Replication

**DOI:** 10.1038/s41598-017-10236-x

**Published:** 2017-08-30

**Authors:** Suzana Aulić, Lara Masperone, Joanna Narkiewicz, Elisa Isopi, Edoardo Bistaffa, Elena Ambrosetti, Beatrice Pastore, Elena De Cecco, Denis Scaini, Paola Zago, Fabio Moda, Fabrizio Tagliavini, Giuseppe Legname

**Affiliations:** 1 Laboratory of Prion Biology, Department of Neuroscience, Scuola Internazionale Superiore di Studi Avanzati (SISSA), Trieste, Italy; 2 Department of Medical, Oral, and Biotechnology Science and Center on Aging Sciences and Translational Medicine (CeSI-MeT) “G. D’Annunzio” University of Chieti-Pescara, Chieti, Italy; 3 Unit of Neuropathology and Neurology 5, IRCCS Foundation Carlo Besta Neurological Institute Italy Laboratory, Milano, Italy; 4 ELETTRA Sincrotrone Trieste S.C.p.A, Basovizza, Trieste Italy; 5 Department of Physics, University of Trieste, Trieste, Italy; 6 Department of Life Sciences, University of Trieste, Trieste, Italy

## Abstract

The precise molecular mechanism of how misfolded α-synuclein (α-Syn) accumulates and spreads in synucleinopathies is still unknown. Here, we show the role of the cellular prion protein (PrP^C^) in mediating the uptake and the spread of recombinant α-Syn amyloids. The *in vitro* data revealed that the presence of PrP^C^ fosters the higher uptake of α-Syn amyloid fibrils, which was also confirmed *in vivo* in wild type (*Prnp* ^+/+^) compared to PrP knock-out (*Prnp* ^−/−^) mice. Additionally, the presence of α-Syn amyloids blocked the replication of scrapie prions (PrP^Sc^) *in vitro* and *ex vivo*, indicating a link between the two proteins. Indeed, whilst PrP^C^ is mediating the internalization of α-Syn amyloids, PrP^Sc^ is not able to replicate in their presence. This observation has pathological relevance, since several reported case studies show that the accumulation of α-Syn amyloid deposits in Creutzfeldt-Jakob disease patients is accompanied by a longer disease course.

## Introduction

α-Synuclein (α-Syn) protein accumulates in the form of Lewy bodies (LBs) and Lewy neurites (LNs) in a group of neurodegenerative diseases (ND) collectively known as synucleinopathies, which comprise Parkinson’s disease (PD), dementia with Lewy bodies (DLB) and multiple system atrophy (MSA)^1, 2^. LBs and LNs accumulate and spread within the central nervous system (CNS)^3, 4^. Aggregated forms of α-Syn are able to recruit and seed the endogenous protein and initiate the spreading throughout cells in cellular and animal models^5–10^. Several mechanisms of cell-to-cell spread have been proposed (endocytosis, receptor-mediated endocytosis, exosomes, tunneling nanotube formation etc. refs 5, 6 and 11–15), and most probably all of them contribute to some extent to α-Syn aggregate propagation. However, little is known about the molecular mechanisms underlying α-Syn propagation. Recently, it has been identified that α-Syn-biotin PFF bind lymphocyte-activation gene 3 (LAG3) and in this way initiates the transmission from neuron-to-neuron^16^. In addition, recent studies show that the cellular prion protein (PrP^C^) may be involved in the pathophysiological processes underlying several ND^17^. Indeed, in this context the PrP^C^ expression is essential for the progression of prion disorders (i.e. Creutzfeldt-Jakob disease) and for the induction of neurotoxicity of amyloid-β (Aβ) in Alzheimer’s disease (AD)^18^. The potential role of PrP^C^ as an Aβ oligomer receptor has been described^19^.

Here we show that in different experimental models, PrP^C^ is able to mediate uptake of α-Syn amyloid fibrils in contrast to models where PrP^C^ expression is ablated. Mouse α-Syn amyloid fibrils are readily endocytosed in the murine neuroblastoma Neuro-2a (N2a) cells expressing functional PrP^C^ compared to cells in which PrP^C^ expression is absent, suggesting that PrP^C^ could in part mediate the internalization process. Furthermore, wild-type mice (*Prnp* ^+/+^) injected with α-Syn amyloid fibrils displayed higher amounts of α-Syn aggregates compared to animals deprived of PrP^C^ (*Prnp* ^−/−^).

Finally, α-Syn amyloid fibrils are able to interfere with prion replication through the binding to PrP^C^. We show here that the possible mechanism behind this phenomenon is explained by the binding of α-Syn amyloid fibrils to PrP^C^ and its ensuing increased processing that leads to the formation of protein fragments that are neuroprotective (N1 and C1 fragments), and able to clear residual PrP^Sc^ deposits.

## Results

### α-Syn amyloid fibrils uptake is facilitated in cells expressing PrP^C^

**Figure 1:**
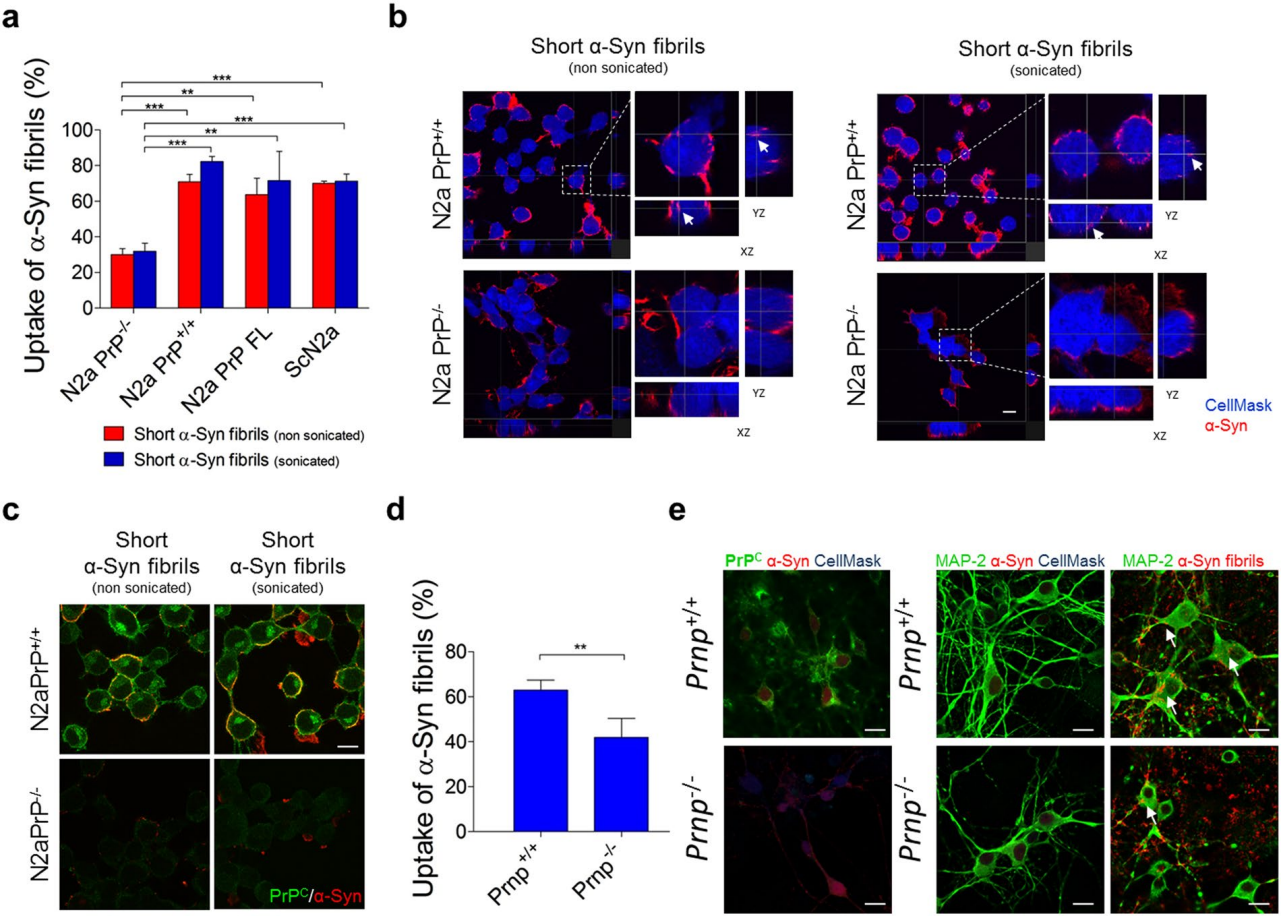
Uptake of mouse α-Syn amyloid fibrils in N2a cells and primary culture of hippocampal neurons. (**a**) Uptake quantification after 24 hours incubation with neuroblastoma (N2a) cells that express and that were ablated for the PrP^C^ expression show that 82.1 ± 2.9% of N2a PrP^+/+^ are able to uptake α-Syn amyloid fibrils in comparison to only 31.8 ± 4.7% of N2a PrP^−/−^ cells. Data are shown as mean ± SD (**P < 0.01, ***P < 0.0001 for two-way ANOVA with Bonferroni’s posttests, *N* = 3 experiments with total of four hundred cells), (**b**) with relative orthogonal views of confocal images. Interaction accounts for 1.21% of the total variance; F = 0.66. (**c**) Non-sonicated and sonicated α-Syn amyloid fibrils (in red) co-localize with the endogenous membrane-bound PrP^C^ (in green) on the surface of neuroblastoma cells, while α-Syn fibrillar species did not even bind plasma membrane of N2a PrP^−/−^ cells. Scale bars 15 μm. (**d**) Uptake quantification after 24 hours incubation with primary cultures of hippocampal neurons deriving from FVB *Prnp* ^+/+^ and FVB *Prnp* ^−/−^ mice. A total of four hundred cells were counted. Data are shown as mean ± SD. Data were evaluated by unpaired Students’ *t*-test. Statistical analysis is indicated as: **P < 0.01. (**e**) Representative images of control and α-Syn fibril-treated hippocampal neurons after 24 hours. On the left panels: in red, α-syn fibrils; in green, PrP^C^; and in blue, nuclei and cytoplasm (CellMask staining). On the right panels: in red, α-syn fibrils; in green, MAP-2; and in blue, nuclei and cytoplasm (CellMask staining). Scale bars represent 10 μm.

To assess whether PrP^C^ expression may facilitate α-Syn amyloid entrance in cells we first used an *in vitro* approach. We compared uptake of recombinant (rec) mouse α-Syn amyloid fibrils in N2a cells that constitutively express PrP^C^ and in the same cell line ablated for PrP^C^ (using CRISPR-Cas9-Based Knockout system)^20^. First, we produced highly pure rec mouse α-Syn protein that was subjected to the fibrillation process (Supplementary Fig. S1a–d). Finally, the resulting mouse α-Syn fibrils were subjected to biochemical analysis (Supplementary Fig. S1e) and the amyloids were structurally characterized by atomic force microscopy (AFM) (Supplementary Fig. S2)^21^. Quantification showed that the sonication process breaks the fibrils into more homogenous smaller species (Supplementary Fig. S2a,b). To investigate whether mouse α-Syn fibrils are internalized by N2a cells we took advantage of confocal microscopy and we quantitatively analyzed the percentage of N2a cells that were able to internalize the amyloids. The data show that 82.1 ± 2.9% of N2a PrP^+/+^ cells had α-Syn aggregates within the cytoplasm compared to only 31.8 ± 4.7% of N2a PrP^−/−^ after 24 h of incubation. Only the removal of PrP^C^ resulted in lower α-Syn uptake, since in N2a PrP^−/−^ cells that were transfected with full-length PrP and in N2a PrP^+/+^ infected with RML prion strain^22^ the uptake was comparable (71.6 ± 16.5% and 71.3 ± 4.0%, respectively, Fig. 1a). The non-sonicated α-Syn amyloids were internalized in similar percentage (Fig. 1a,b). In addition, both sonicated and non-sonicated mouse α-Syn amyloid preparations bound to the PrP^C^ on cell membrane, whereas in N2a PrP^−/−^ the interaction was hampered (Fig. 1c). Reintroduction of full-length PrP into N2a PrP^−/−^ cells rescued this effect (Supplementary Fig. S3). After addition of α-Syn amyloids PrP^C^ levels slightly increased, but in four subsequent serial passages PrP^C^ levels were maintained at basal levels (Supplementary Fig. S4a,b). However, this slight increase of protein levels was not due to the mRNA increase since levels of *Prnp* transcripts were not altered after treatment (Supplementary Fig. S4c). The viability assay sowed that the exposure to 2 μM exogenous α-Syn amyloids (for 24 hours) did not altered significantly the percentage of viable cells (Supplementary Fig. S5). Indeed, numerous studies show that the cytotoxicity of α-Syn fibrils and protofibrils remains controversial in relation to their roles in neurodegeneration. In PrP^C^ expressing cells α-Syn fibrils were predominantly found in lysosomal vesicles (Supplementary Figs S6 and S7). Lysosomes are the common degradative end-point at which the endosomal pathway converges; this is the reason why in certain percentage of cells we can still observe α-Syn amyloids in endosomes (Supplementary Fig. S7, upper panel).

We next investigated α-Syn amyloid internalization in primary cultures of hippocampal neurons. We used both PrP wild-type and knock out mice (FVB *Prnp* ^+/+^ and *Prnp* ^−/−^). In FVB *Prnp* ^+/+^ mice 62.9 ± 4.6% of neurons internalized α-Syn non-sonicated fibrils (after 24 hours of incubation), while the internalization in *Prnp* ^−/−^ neurons was less efficient (41.9 ± 8.5%, Fig. 1d,e). Taken together these results indicate that PrP^C^ is required for the internalization of α-Syn fibrils.

### Recombinant PrP binds α-Syn amyloids

It has been reported that PrP^C^ binds amyloids like PrP^Sc^ and Aβ^19^. Since confocal microscopy experiments revealed the co-localization between PrP^C^ attached to the cell membrane and exogenously added α-Syn amyloids (Fig. 1c) we moved on to better characterize the molecular interaction between the two proteins. In this context, enzyme-linked immunosorbent assay (ELISA) and surface plasmon resonance (SPR) experiments were performed. The data reveal that PrP^C^ binds fibrillary α-Syn *in vitro* (Supplementary Fig. S8b,c). Both rec full-length mouse PrP MoPrP(23-231) and truncated MoPrP(89-231), bind rec α-Syn amyloids. We do not observe a linear ELISA signal increasing of the ratio between the two proteins considering that we have an non-homogeneous population of α-Syn aggregates. Additionally, ELISA signal increases with higher concentrations of monomeric α-Syn in the case of interaction with both rec PrP (full-length and truncated). More precisely, we observed that the N-truncated rec PrP binds more weakly α-Syn fibrils. On the contrary, in the full-length rec PrP 1:3 dilution ratio led to a higher binding of the two proteins. These data led us conclude that the binding to the PrP occurs mainly at the N-terminal part of the protein. This is in agreement with several studies that report the N-terminal part of prion protein as affinity site for amyloid-like structures (Aβ, PrP^Sc^)^19^.

Furthermore, taking advantage of SPR, we were able to calculate binding constants. To quantify the binding we first immobilized sonicated fibrils on the surface of one flow cell of a CM5 biosensor chip and added monomeric α-Syn protein (Supplementary Fig. S9a). Supplementary Fig. S9b shows the binding of immobilized sonicated fibrils with rec full-length MoPrP(23-231). Two K_D_ values (3.1 nM and 36.5 nM) were determined by the ratio between the two k_*off*_ values obtained and k_*on*_. These K_D_ values suggest that: (i) PrP - α-Syn amyloid binding occurs forming first weak interactions and then stronger interactions, and/or (ii) smaller α-Syn species establish stronger interactions with PrP while longer α-Syn amyloid fibrils form weaker interaction. The latter explanation seems more plausible since α-Syn amyloid population is not homogeneous.

### Detection of Proteinase K-resistant α-Syn deposits and other hallmarks of synucleinopathy in *Prnp*^+/+^ and *Prnp*^−/−^ mice

**Figure 2:**
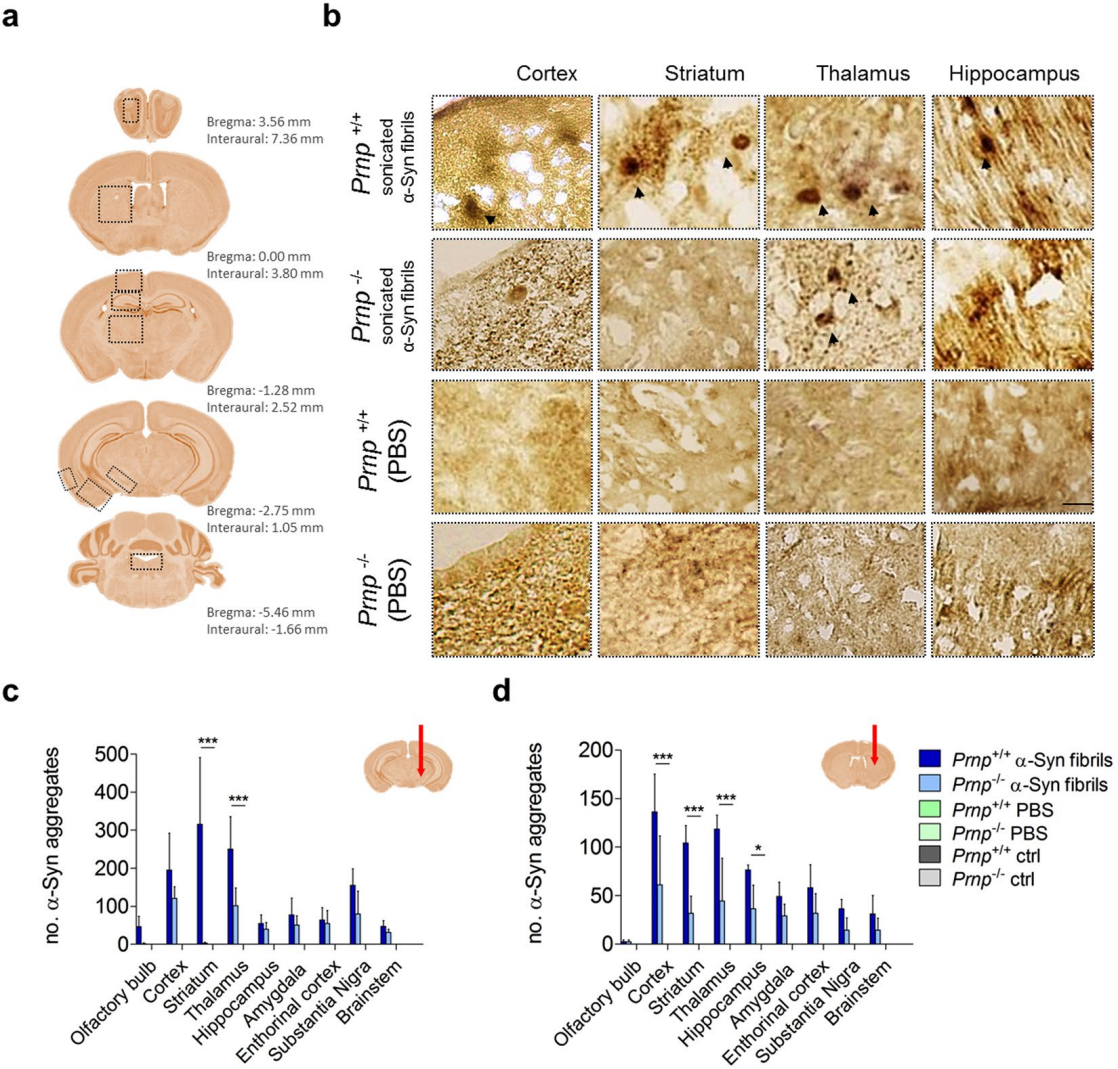
Stereotaxic inoculation of sonicated α-Syn amyloid fibrils seeds the aggregation of endogenous mouse α-Syn in FVB mice. (**a**) Four different levels of CNS were considered for the counting of α-Syn deposits (olfactory bulb; striatum; motor cortex, M1, M2; hippocampus CA1, CA2, CA3; thalamus; amygdala; *Substantia nigra*; enthorinal cortex; brainstem). (**b**) Accumulation of PK-resistant α-Syn deposits in striatum, cerebral cortex, thalamus, and hippocampus in mice injected in *Substatia nigra*. Scale bar 50 μm. (**c**,**d**) Quantification of PK-resistant α-Syn deposits in all considered brain areas show that *Prnp* ^+/+^ FVB mice are able to accumulate more α-Syn deposits compared with *Prnp* ^−/−^. *Prnp* ^+/+^ FVB mice accumulate more PK-resistant α-Syn when injected in the *Substantia nigra* (**c**), or in the striatum (**d**) compared to *Prnp* ^−/−^. Data are represented as mean ± SD, for two-way ANOVA with Bonferroni’s posttests, *N* = 3 animals per group. For (**c**) interaction accounts for 27.82% of the total variance; F = 4.86. The *P* value is < 0.0001. For (**d**) interaction accounts for 22.09% of the total variance; F = 5.57. The *P* value is < 0.0001). Red arrows show injection sites.

*Prnp* ^−/−^ mice neither propagate prions nor develop scrapie suggesting the central role of PrP^C^ in the development of prion diseases^23, 24^. The critical feature for the development of prion disease is a direct interaction of PrP^C^ with PrP^Sc^ which acts as a template for the conversion^25^. Additionally, PrP^C^ might be implicated in the pathogenesis of AD. Lauren *et al*. reported that PrP^C^ could mediate Aβ toxicity^18^. Several studies report the direct interaction between PrP^C^ and β-sheet rich species like Aβ-oligomers^26–28^. However, there are no reports of role of PrP^C^ in synucleinopathies. We therefore assessed whether our *in vitro* results might be recapitulated in *in vivo* mouse models. Thus, we performed stereotaxic injections of α-Syn amyloid fibrils in *Prnp* ^+/+^ and *Prnp* ^−/−^ FVB mice both in the *Substantia Nigra pars compacta* (SNpc) and in the striatum. Quantification of DAB-stained sections revealed presence of PK-resistant α-Syn aggregates in *Prnp* ^+/+^ and *Prnp* ^−/−^ mice 5 months post injection (mpi) (Fig. 2).

Injection of α-Syn amyloid fibrils in mice induced the formation of LB-like aggregates in different brain areas (Fig. 2a). In agreement with *in vitro* results, the data show that in general *Prnp* ^−/−^ mice accumulate less PK-resistant α-Syn aggregates in all areas analyzed (Fig. 2c,d). Notably, when *Prnp* ^+/+^ mice were injected within the SNpc, α-Syn aggregates were significantly higher (Fig. 2c). More precisely, we observed an almost complete absence of α-Syn aggregates in the striatum of mice that do not express PrP^C^. While, the mapping of PK-resistant α-Syn deposits in *Prnp* ^+/+^ mice revealed the presence of α-Syn aggregates in the cortex, striatum, thalamus and hippocampus (Fig. 2b). In *Prnp* ^−/−^ mice in all brain areas considered, α-Syn aggregates accumulate less (Fig. 2c). Phosphate-buffer saline (PBS) injections did not result in α-Syn aggregates accumulation in the two groups of animals. PK-resistant α-Syn was absent also in control animals.

Similarly, the stereotaxic injections in the striatum led to the formation of α-Syn aggregates in the brain. However, *Prnp* ^−/−^ mice accumulated lower amount of aggregates compared to *Prnp* ^+/+^ mice (Fig. 2d). In the *Prnp* ^−/−^ mice, the number of α-Syn aggregates was significantly lower in four distinct brain areas (cortex, striatum, thalamus and hippocampus) (Fig. 2d). Generally, *Prnp* ^+/+^ and *Prnp* ^−/−^ animals inoculated within the striatum accumulated less α-Syn aggregates compared to those injected within the SNpc. In both cases the α-Syn-positive LB-like deposits were mainly ipsilateral; still, several α-Syn aggregates were present also in the contralateral regions to the injection site. α-Syn amyloid fibril injection in the SNpc induced strong front and hindlimb clasping in *Prnp* ^+/+^ mice (3/3), while in the case of injection within the striatum only one *Prnp* ^+/+^ mouse was clasping. On the contrary, clasping was never observed in *Prnp* ^−/−^ or control mice (0/3) (Supplementary Fig. S10).

**Figure 3:**
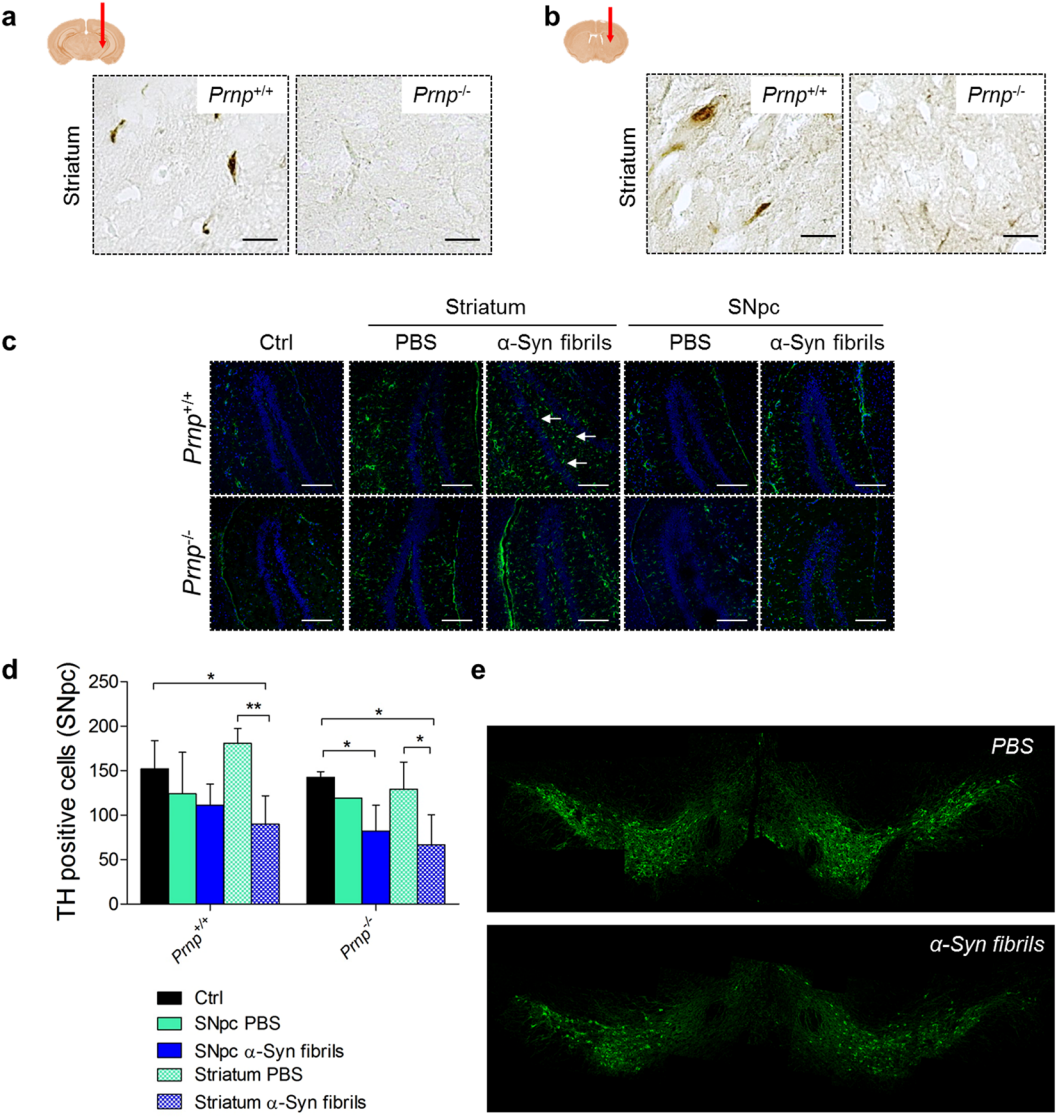
Stereotaxic inoculation of sonicated α-Syn amyloid fibrils seed the aggregation of phosphorylated α-Syn in the CNS, leading to astroglia activation and loss of DA neurons in the *Substantia nigra* (5 mpi). (**a**,**b**) Immunohistochemical staining with an antibody against phosphorylated α-Syn revealed the presence of pathologic α-Syn deposits in the striatum of mice inoculated in the *Substantia nigra*, and in the striatum. Red arrows show the injection sites. Scale bars 25 μm. (**c**) Representative images of GFAP-immunostained samples show that there is a higher astroglial activation in α-Syn inoculated mice compared to PBS-inoculated or non-inoculated controls. Scale bars 100 μm. (**d**) Tyrosine hydroxylase (TH) immunoreactivity quantification in *Substantia nigra* neurons show that seeded α-Syn pathology leads to loss of DA neurons after nigral or striatal inoculation. Values are given as means ± SD. Statistical significance was calculated by using two-way ANOVA followed by Turkey’s test. *P < 0.05, **P < 0.01, ***P < 0.001. (**e**) Representative mosaic images that cover *Substantia nigra pars compacta* and Ventral Tegmental Area (VTA) of WT mice inoculated with PBS (upper panel) or with α-Syn fibrils (lower panel) in the striatum.

Another hallmark of synucleinopathies is the presence of phosphorylated α-Syn deposits at residue S129 (pS129)^29^. Immunohistochemical analysis for pS129-α-Syn revealed the presence of accumulation of phosphorylated α-Syn aggregates in both *Prnp* ^+/+^ and *Prnp* ^−/−^ mice (Fig. 3a,b).

α-Syn amyloid injection and the ensuing accumulation were accompanied by astrogliosis that was more prominent in *Prnp* ^+/+^ compared to *Prnp* ^-/-^ mice (Fig. 3c). In addition, α-Syn aggregates deposition was accompanied by the gradual loss of tyrosine hydroxylase (TH) immunoreactivity, suggesting that α-Syn accumulation is linked to loss of DA neurons (Fig. 3d).

### α-Syn amyloid fibrils lower PrP^Sc^ levels in scrapie-infected cells and in PMCA assay

**Figure 4:**
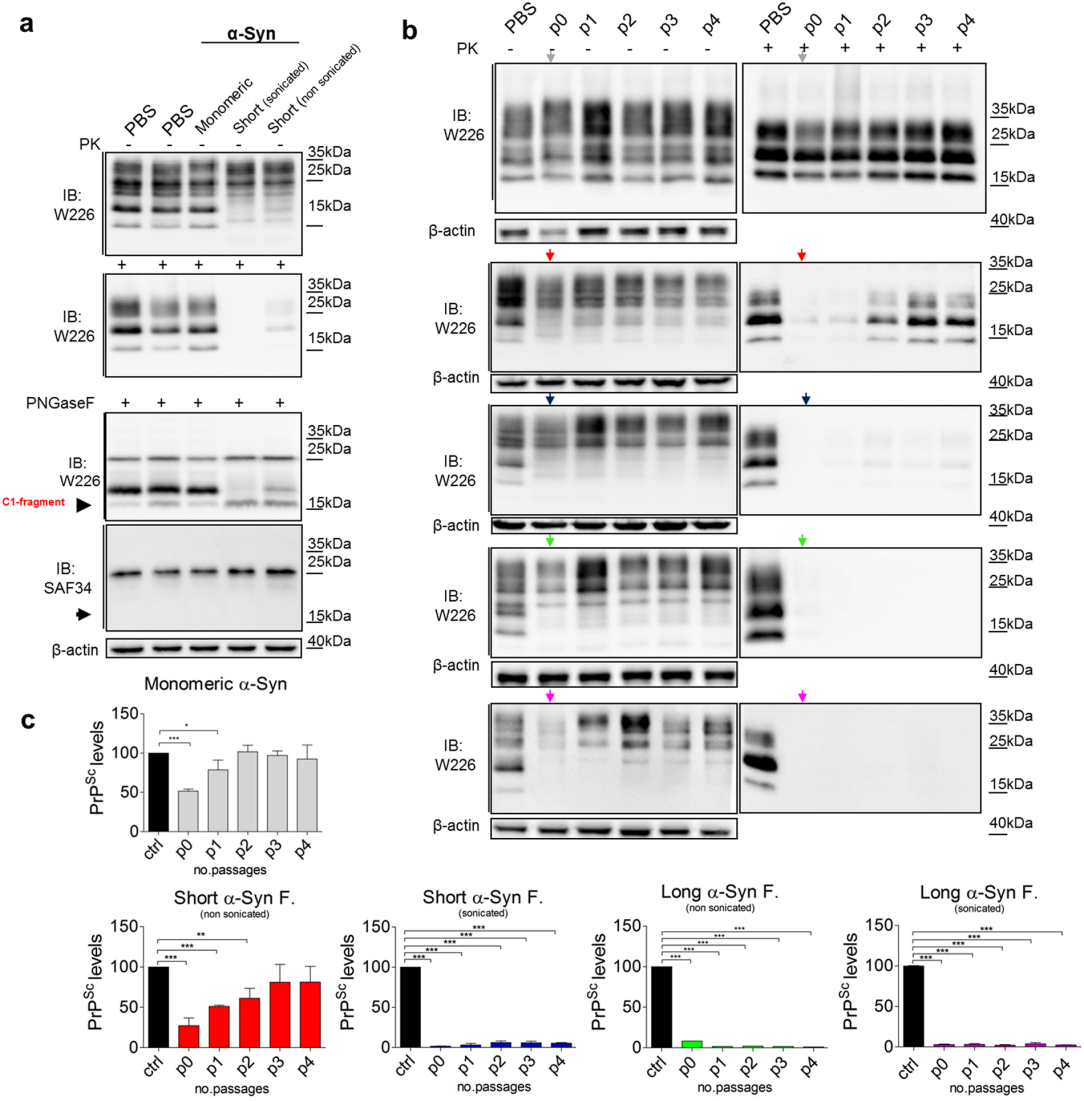
Clearance of PrP^Sc^ from RML prion-infected cells in the presence of α-Syn amyloids. (**a**) PrP^Sc^ levels in ScN2a cells were measured by immunoblotting after four days of culture in the presence of different α-Syn preparations (monomeric and fibrillar α-Syn). (**b**) Western blot analysis of PK-resistant PrP^Sc^ in ScN2a cell lysates after treatment with monomeric α-Syn (grey arrow) or different α-Syn amyloids (short non-sonicated fibrils, red arrow; short sonicated fibrils, blue arrow; long non-sonicated fibrils, green arrow; long sonicated fibrils, magenta arrow) over four serial passages. (**c**) Quantification of three independent experiments. The values are shown as a percentage of PK-resistant form relative to total PrP/β-actin. β-Actin is a loading control. Data are represented as mean ± SD. Data were evaluated by unpaired Student’s *t*-test. Statistical analysis is indicated as: *P < 0.05, **P < 0.01, ***P < 0.001.

**Figure 5:**
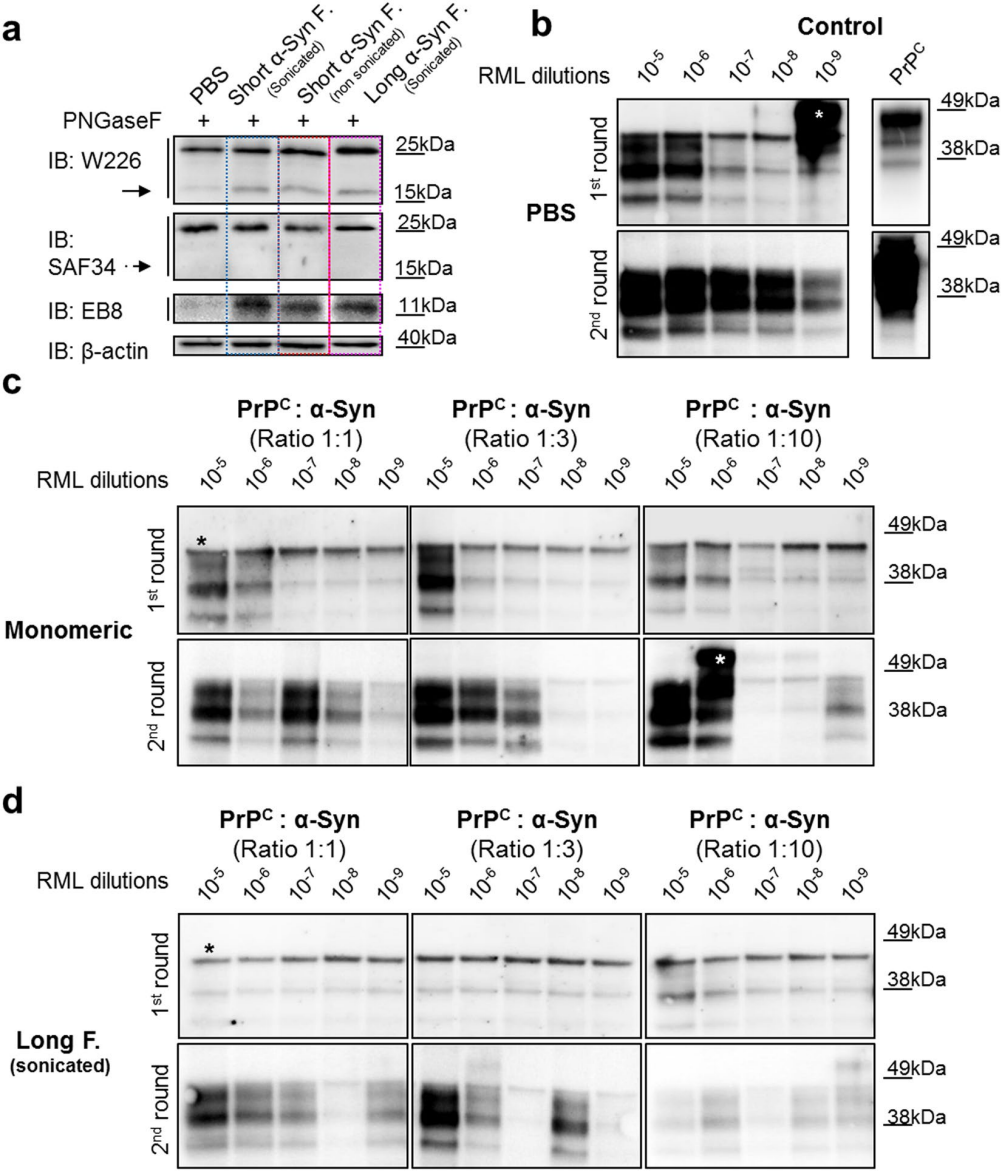
Increased processing of the cellular PrP after treatment with mouse α-Syn amyloids, and the role of fibrils and monomeric α-Syn on cyclic amplification of PrP^Sc^. (**a**) After PNGase F deglycosylation cell lysates of N2a cells treated with different α-Syn prep were separated by SDS-PAGE and PrP was immunoblotted with two Abs that recognize C-terminal part (W226 upper membrane), and N-terminal of the prion protein (SAF34 lower membrane), showing increased levels of α-cleavage product, C1 fragment. While, the immunoprecipitation of the medium with N-terminal Ab EB8 after four days of treatment show the other counterpart released within the medium, N1 fragment. β-Actin is a loading control. (**b**–**d**) Effect of α-Syn amyloid fibrils on conversion of PrP^Sc^ in two rounds of PMCA show the inhibition effect compared to control conditions where the monomeric α-Syn was used or only a buffer (PBS). The number of cycles of incubation-sonication and the ratios α-Syn fibrils: PrP^C^ are indicated. * = unspecific binding (white asterisk: uncomplete PK digestion).

Our data illustrate the role of PrP^C^ in facilitating the internalization of α-Syn amyloids. Several studies report the importance of expression of PrP^C^ in prion pathology^30, 31^. However, the role of PrP^C^ in mediating PrP^Sc^ formation and its spread in the presence of α-Syn amyloids has never been described. Indeed, in two different assays of prion replication we observed lower PrP^Sc^ levels when recombinant α-Syn amyloids were added (Figs 4 and 5). More precisely, the supplement of α-Syn amyloids to cell culture that replicates the RML prion strain (ScN2a cell line) clear PrP^Sc^ levels after four days of incubation. In addition, through four subsequent passages no prions were detected. The treatment with various α-Syn amyloids decreased the PrP^Sc^ levels to different extent (Fig. 4a). Both, long non-sonicated α-Syn amyloids and those sonicated for 5 minutes had the most efficient inhibitory effect on prion replication in four further passages (Fig. 4b,c, Supplementary Fig. S11 confirms the inhibition also in immunofluorescence experiments). Importantly, in this context, it is worth noting that the monomeric α-Syn was not able to inhibit prion replication through passaging (Fig. 4b, uppermost panel). In order to check if this propriety was characteristic of only mouse α-Syn sequence we treated the ScN2a cells also with human WT α-Syn and all PD-related mutants. Human α-Syn sequence differs from mouse sequence in seven amino acids. These differences reflect in lag phases of fibril formation (all characterizations of human α-Syn proteins are reported in Supplementary Figs S12 and S13). Despite these differences in primary sequence, all human α-Syn amyloid proteins (WT and PD-related mutants) retain their PrP^Sc^ inhibiting properties, resembling mouse α-Syn amyloid protein (Supplementary Fig. S14b,c).

We checked also whether other amyloid forming proteins (like Aβ and tau fragment K18)) were able to clear PrP^Sc^ levels in ScN2a cells (Figs S15 and S16). Although, it is widely reported that Aβ binds PrP^C^ still, it is not able to clear PrP^Sc^ in cell culture (Fig. S16).

We find that the clearance of PrP^Sc^ aggregates in cell culture is most likely due to the formation of C1 fragment together with N1 fragment (Fig. 5a). C1 and N1 fragment formation is a result of increased α-cleavage processing of PrP^C^ in the presence of α-Syn amyloids. This inhibitory effect on prion replication of mouse α-Syn amyloids was further analyzed also by a cell-free technique such as Protein Misfolding Cyclic Amplification (PMCA)^32, 33^. In particular, we assessed α-Syn amyloids ability to block prion replication using brain homogenate of *Prnp* ^+/+^ FVB mice as a substrate and RML prion strain as a seed. The results show that in PMCA assay the α-Syn amyloid presence (in three different molar concentrations, 1:1, 1:3 and 1:10) reduced the rate of RML replication in two serial rounds of PMCA (Fig. 5d), while the replication of prions was present in control conditions (Fig. 5b,c).

Amyloid seeding assay (ASA)^34^ confirmed the data observed in ScN2a cells, and in PMCA experiments (Supplementary Fig. S17).

## Discussion

Several independent studies described the accumulation of α-Syn pathology after intracerebral inoculation of recombinant α-Syn fibrils in wild type^9, 10^, and in transgenic mice^7, 35, 36^. However, the mechanisms through which exogenously injected fibrils enter the cells and through which insoluble α-Syn is transported to other neurons remain unknown. In this study we provide new insights of how the expression of PrP^C^ in neurons may facilitate the accumulation and spreading of α-Syn aggregates. First, we found that higher percentage of N2a cells, that endogenously express PrP^C^, internalize α-Syn amyloid fibrils compared to N2a cells that were ablated for PrP^C^. Consistent with these observations, we observed higher internalization rate in primary cultures of hippocampal neurons deriving from FVB *Prnp* ^+/+^ mice compared to *Prnp* ^−/−^ neurons. These findings suggested that PrP^C^ may bind α-Syn amyloids and thus accumulate it in the cytoplasm. Indeed, in biochemical and biophysical experiments we were able to show that this molecular interaction occurs. Our *in vitro* observations were also confirmed by *in vivo* experiments, where the *Prnp* ^+/+^ mice presented abundant α-Syn pathology compared to *Prnp* ^−/−^ FVB mice, after intracerebral injection of α-Syn amyloids. Furthermore, *Prnp* ^+/+^ mice showed increased levels of other key markers linked to LB pathology. Therefore, the potential function of PrP^C^ in binding and mediating cell-to-cell spread of α-Syn amyloids offers an intriguing new role for PrP^C^ and opens new venues of research.

However, scrapie load in cell culture decreased in α-Syn amyloid presence. The inhibitory effect we observed is most readily explained by α-Syn amyloid binding to PrP^C^ on the cell surface, which blocks the docking of PrP^Sc^ template for further replication. Indeed, scrapie prions compete to bind PrP^C^, and we show here that this is also the case for α-Syn amyloids. On the other and the clearance of the pre-existent prions in cell culture could be explained by the increased processing of PrP^C^ in the presence of α-Syn amyloids. α-Cleavage of PrP^C^ is a process that yields two neuroprotective fragments^37, 38^, C1 fragment (18 kDa, after deglycosylation runs at 16 kDa) that accumulate at the plasma membrane, and N1 fragment (11 kDa) that is released in the medium. This processing has been shown to regulate physiological function of the prion protein generating biologically active fragments that can even modulate the course of prion progression. The latter is also shown to interfere with the neurotoxicity of Aβ-oligomers. In this study we show that the amount of this soluble N-terminal fragment can be involved also in the α-Syn amyloid presence. Indeed, confirming our hypothesis the treatment with Aβ didn’t increased PrP^C^ processing since we did not observe any formation of the two neuroprotective inhibitory fragments (N1 and C1) and most importantly we could not observe any effect on PrP^Sc^ levels in its presence, like in the case of α-Syn amyloid treatment (Fig. 5a). On the other hand, the K18 fragment amyloids of tau protein (consisting of four repeats in the C-terminal half of the protein; all biochemical characterizations of tau fragment is reported in Supplementary Fig. S16) are able to increase the PrP^C^ processing forming the inhibitory fragments (C1 and N1). Accordingly, K18 amyloids retain inhibiting capacity on scrapie aggregates in cell culture (Supplementary Fig. S16c). Indeed, we observed the increased formation of C1 fragment bound to the cell membrane and the N1 fragment released in the medium after treatment with α-Syn and K18 amyloids (Fig. 5a, and Supplementary Fig. S16f).

The present study indicates that PrP^C^ might contribute to enhanced binding of α-Syn fibrils to the plasma membrane, in line with other studies reporting an increased level of binding between Aβ and PrP^C^ during aging in several mouse models of Alzheimer’s disease. From the clinical point of view, there are several case reports describing the coexistence of prions and α-Syn deposits in sporadic (sCJD) and variant CJD (vCJD)^39–43^. When LBs develop in CJD patients the course of the disease is longer (3–4 years) compared to classical sCJD and vCJD (less than 1 year). In all described case reports, patients accumulate LBs and LNs in *Substantia nigra* and in cerebral cortex. Most likely, prion protein expressed at distinct levels of the CNS binds LBs and LNs and contributes to the cell-to-cell spread of the α-Syn pathology in CJD patients. At the same time, in these conditions the increased processing of the physiological PrP produces neuroprotective fragments that clear prion deposits. Consequently, the course of the CJD pathology is much longer in those patients that develop LB aggregates compared to those without LB deposits.

The observation that α-Syn amyloids can bind PrP^C^ may have potential therapeutic application. In fact, antibodies raised against PrP^C^ are able to block prion replication efficiently by interfering with PrP^C^–PrP^Sc^ interaction^44^. The same could be predicted for α-Syn pathology, antibodies against PrP^C^ could prevent α-Syn amyloids binding to PrP^C^, thus precluding cell entry and spreading of synucleopathies.

## Methods

### Fibrillation of mouse α-Syn

Prior to fibrillation, the protein was filtered with 0.22 μm syringe filter and the concentration was determined by absorbance measured at 280 nm. Purified mouse α-Syn (1.5 mg/mL) was incubated in the presence of 100 mM NaCl, 20 mM Tris-HCl pH 7.4 and 10 μM ThioflavinT (ThT). Reactions were performed in black 96-well plates with a clear bottom (Perkin Elmer), in the presence of one 3-mm glass bead (Sigma) in a final reaction volume of 200 μL. Plates were sealed and incubated in BMG FLUOstar Omega plate reader at 37 °C with cycles of 50 sec of shaking (400 rpm, double-orbital) and 10 sec of rest. ThT fluorescence measurements (excitation: 450 nm, emission 480 nm, bottom read) were taken every 15 min.

For cell culture experiments, fibrillation of the protein was performed as described above, but in the absence of ThT. After fibrillation, the reaction mixtures were ultracentrifuged for 1 h at 100,000 g (Optima Max-XP, Beckman) and re-suspended in sterile PBS, aliquoted and stored at −80 °C until use.

### Cell culture

Mouse neuroblastoma N2a cells were kindly provided by Prof. Chiara Zurzolo (Unité de traffic membranaire et pathogenèse, Institute Pasteur, Paris, France). ScN2a cells are clones persistently infected with the RML prion strain as described by Prusiner’s group *(20)*. Cells were grown and maintained at 37 °C/5% CO_2_ incubator in minimal essential medium (MEM) + glutamax (Thermo Fisher Scientific Inc.), supplemented with 10% fetal bovine serum, 1% non-essential aminoacids, and, 100 units/mL penicillin and 100 μg/mL streptomycin. 30000 cells/dish were cultured for treatments. N2aPrP^−/−^ cells were kindly provided by professor Gerold Schmitt-Ulms (Tanz Centre for Research in Neurodegenerative Diseases, University of Toronto, Toronto, Ontario, Canada), for which they used the CRISPR-Cas9-Based Knockout system to ablate the expression of PrP protein (19).

Primary cell cultures were prepared from hippocampal of mice FVB WT and KO. Wild-type FVB and *Prnp* KO strains mice used in this study were housed at the SISSA mouse facility. Animals handling and subsequent procedures were in accordance with European [European Communities Council Directive of November 24, 1986 (86/609/EEC)] and Italian laws (D.L. 04.03.2014, n°26) and were approved by SISSA Board for Animal Welfare.

### Primary neuronal cultures

Hippocampi were dissected from 0–1-day-old postnatal animals. The isolated tissue was quickly sliced and digested in a digestion solution containing Trypsin (Sigma-Aldrich) and DNAse (Sigma-Aldrich). The reaction was stopped with Trypsin inhibitor (Sigma-Aldrich) and cells were mechanically dissociated in a dissection medium containing DNAse. After centrifugation, the cell pellet was resuspended in the culture medium and distributed in a 12 well Multiwell (Falcon), on coverslips (12 mm diameter) previously coated with polyornithine (50 μg/mL, Sigma-Aldrich) and Matrigel (2% (w/v), BD). Plating was carried out at a density of 100.000 cells per coverslip.

Hippocampal neurons cultures were incubated at 37 °C, in a humidified atmosphere with 5% CO2 in culture medium, consisting of MEM (Gibco), supplemented with 35 mM glucose (CarloErba Reagents), 1 mM Apo-Transferrin, 15 mM HEPES, 48 mM Insulin, 3 mM Biotin, 1 mM Vitamin B12 (Sigma-Aldrich) and 500 nM Gentamicin (Gibco) and 5–10% dialyzed FBS (Gibco).

### Western blotting

After 4 days of treatment with different α-Syn preparations, medium was removed and the cells were washed twice with PBS 1X and lysed in lysis buffer. Total protein content of cell lysates was measured using bicinchoninic acid protein (BCA) quantification kit (Pierce) and stored at −20 °C until analysis. The total of 30 μg/mL of cell lysates were resuspended in Laemmli loading loading buffer, and boiled for 10 min at 95 °C. Subsequently the samples were loaded onto a 12% Tris-Glycine SDS-PAGE gel, and transferred onto nitrocellulose membrane (GE Healthcare), blocked using 5% non-fat milk (w/v) blocking solution for 1 h at room temperature with agitation followed by incubation with anti-PrP antibodies (W226, 1:1000; SAF43, 1:1000) or anti β-actin (1:50000, A3854 Sigma-Aldrich) diluted in blocking solution. Membranes were washed with TBST (0.1% Tween 20 in TBS), and incubated in horseradish–peroxidase-conjugated (HRP) goat anti-mouse secondary Ab (diluted 1:2000) for 1 h. The membranes were washed in TBST and proteins were visualized following the manufacturer’s instructions using Amersham ECL Western Blotting Detection Reagent (GE Healthcare) with UVITEC Cambridge. Quantitative densitometry analysis of proteins was performed using NIH Image software (ImageJ 1.50a, USA).

### Immunofluorescence

Thirty thousands cells were cultured on coverslips and treated with α-Syn fibrils (2 μM), afterwards the cells were fixed with 4% formaldehyde in PBS for 30 min. Cells were then washed three times with PBS (1X) followed by blocking in 5% Normal Goat Serum (NGS, ab7481, Abcam)/0.3% Triton X-100 for 1 h, at room temperature (R.T.) Cells were incubated with primary antibodies diluted in 1% of blocking buffer (anti-PrP Ab W226, 1:500, anti-α-Syn Ab C-20-R, Santa Cruz, 1:1,000), followed by three washings with PBS and secondary antibody incubation (goat anti-mouse Alexa488, and goat anti-rabbit Alexa594, Life Technologies). To ensure that α-Syn preparations were within the cell cytoplasm a specific dye that labels the entire cell was used (HCS CellMask™ dye). Cells were mounted in Aqua Poly/Mount (Polysciences), and images were acquired using Leica confocal microscope (Leica TCS SP2, Wetzlar, Germany). Quadruple staining was carried out using 4% paraformaldehyde fixed cells. Non-specific protein interactions were blocked with 10% normal goat serum (Sigma) and 0.3% Triton-X100 and incubated with the primary antibodies (D18 for PrP^C^, C-20-R for αSyn, from Santa Cruz, EEA1-endosomal, Calenexin-endoplasmatic reticulum, Lamp1-lysosomal and M6PR-Golgi markers, from Abcam), in a humidified chamber at 4 °C overnight. Following washes in PBS the cells were incubated with secondary antibodies conjugated to biotin (1:500, ThermoFisher) followed by incubation with Alexa Fluor 647 Streptavidin conjugate (1:500, ThermoFisher). Coverslips were mounted in Aqua Poly/Mount (Polysciences), and images were acquired using C1 Nikon confocal microscope. Hippocampal neurons grown for 6 days *in vitro* (DIV), were fixed with 4% paraformaldehyde/PBS and immuno-stained with monoclonal MAP-2 antibody (Abcam), anti α-Syn antibody (C-20-R, Santa Cruz). Followed by the secondary antibody incubation (goat anti-mouse Alexa488, and goat anti-rabbit Alexa594, Life Technologies) and HCS CellMask™ dye (Thermo Fisher Scientific). Cells were mounted in Aqua Poly/Mount (Polysciences), and images were acquired using C1 Nikon confocal microscope.

### Uptake quantification

The uptake quantification was performed in blind using Oil Immersion 63X objective on more than 200 cells per one single independent experiment (in total of *N* = 3). Random fields per coverslips at 63x magnification were captured using Leica confocal microscope (Leica TCS SP2, Wetzlar, Germany). To observe internalized α-Syn fibrils, the coverslips were double-labeled with anti- α-Syn antibody and whole cytoplasmic dye CellMask. Cells considered α-Syn positive were those in which the aggregates were found in perinuclear zone. The images were acquired as 20–30 z-stacks of 0.22 μm, 1024 × 1024, and analyzed using Orthogonal Views function in Image J (NIH). Data are represented as % of total cell counted in three independent experiments.

### Protein Misfolding Cyclic Amplification assay (PMCA)

PMCA was performed as previously described (33). Briefly, as a substrate, we used brain specimens obtained from outbred FVB animals. Brains were harvested after perfusion, and 10% homogenate was prepared in conversion buffer (PBS containing 150 mM sodium chloride and 1% Triton X-100) with the addition of protease inhibitors. Different concentrations of α-Syn fibrils and α-Syn monomers were added to the substrate. PBS was used as control for the reaction. To evaluate the inhibitory effect of α-Syn (monomers vs fibrils) RML prion strain were spiked at different dilutions (from 10^−5^ to 10^−9^) and assessed by means of PMCA. The reaction mix were transferred in 0.2 mL tubes, positioned on an adaptor placed on the plate holder of a microsonicator (Misonix, Model S3000) and subjected to 96 cycles of PMCA. Each cycle (also referred as PMCA round) consisted of 29 min and 40 sec of incubation at 37/40 °C followed by a 20 sec pulse of sonication set at potency of 260–270 Watt. After one round of PMCA, an aliquot of the amplified material was diluted 10-folds into fresh substrate and a further PMCA run performed following the same procedure. To increase PMCA efficiency, teflon beads (n = 3) were added to the samples before each round of amplification. To avoid samples cross-contamination between each round, thorough decontamination of instruments and equipment was performed using 2 N sodium hydroxide (NaOH) or 4 M guanidinium-hydrochloryde.

### *In vivo* Experimental design

#### Stereotactic surgery

Mice were subdivided into groups composed of 3 animals each and intraperitoneally anesthetized with a mixture of Xylazine (15 mg/kg) and Zoletil (15 mg/kg). Sonicated α-Syn short fibrils (15 μg) or sterile saline solution were stereotactically injected via a 10 μL Hamilton syringe into the Substantia Nigra pars compacta (AP −3.2, ML −1.2, DV −4.4 from Bregma) or in the striatum (AP + 0.2, ML −2, DV −2.4 from Bregma) of the right hemisphere at a rate of 3 μL for 1 min, 3 μL for 2 min, 4 μL for 5 min. The needle was withdrawn of one coordinate and left for further 2 min before being totally removed. After recovery from surgery, animals were regularly monitored and sacrificed at 5 months-post-inoculation (mpi) by an overdose of Xylazine/Zoletil and transcardially perfused with 4% paraformaldehyde (PFA, pH 7.4). Brains were post fixed ON in PFA and sunk in 30% sucrose prior to be embedded in the Killik medium (W01030799, Bio-Optica) and stored at −80 °C until use.

#### Immunohistochemistry

Brains were cut with the Microm 550 cryostat to generate series of 10 µm slides thick coronal sections on Superfrost glass slides (Menzel-Gläser Adhesion Slides SuperFrost® Plus). Endogenous peroxidase inactivation was performed in 3% H2O2, 10% methanol in PBS for 10 minutes. For α-Syn detection, 5 μg/mL of Proteinase-K (PK) digestion was used to reveal aggregates (30 min at room temperature). Blocking was performed in 0.05% Triton-X100, 5% normal goat serum (NGS, Sigma-Aldrich), 1% bovine albumin serum (BSA, Sigma-Aldrich) in PBS. Primary anti-α-Syn antibody (C20-R, Santa Cruz, 1:500) was incubated overnight. For phosphorylated α-Syn (p-α-Syn) detection, slides were previously treated with 70% formic acid for 30 min. Anti-phosphorylated Ser129 α-Syn antibody (P-Syn/81 A, BioLegend, 1:700) was incubated overnight. Sections were then incubated with proper biotinylated-secondary antibodies (Sigma-Aldrich) followed by the VECTASTAIN® ABC Kit. Antibody labeling was revealed using 3′-diaminobenzidine (DAB; Sigma-Aldrich, SIGMAFAST™) as a chromogen. Slides were dehydrated as follow: 1 min in EtOH 50%, 1 min in EtOH 70%, 1 min in EtOH 90%, 1 min in EtOH 100%, 1 min in EtOH/Xilene (1:1), 2 min in Xilene, and mounted with Eukitt mounting medium (Bio Optica). Quantification of PK-resistant α-Syn aggregates was performed with ImageJ software (ImageJ 1.50a).

#### Immunofluorescence, image acquisition and TH count

Brain slices were blocked in 5% NGS, 1% BSA, 1% Triton-X100 in PBS and incubated ON with the primary antibody anti Tyrosine Hydroxylase (TH, ab112, 1:1000) or anti Glial Fibrillary Acidic Protein (GFAP, ab7260, 1:1000). Antibody staining was revealed after incubation with the appropriate secondary antibody Alexa 488 (Life Technologies, 1:500). 4′,6-diamidino-2-phenylindole (DAPI, Sigma-Aldrich, SIGMAFAST™) was used for nuclear staining. Slides were coverslipped with VECTASHIELD Antifade Mounting Medium (H-1000, Vector Laboratories). Fluorescent images (1024 × 1024 pixels) were acquired with the C1 Nikon confocal. For the GFAP fluorescence a 20X objective was used and stacks of z-sections with an interval of 0.25 µm were sequentially scanned, to obtain representative images of the hippocampus. The same protocol was used for the reconstruction of the *Substantia nigra* - Ventral Tegmental Area as representative image. TH labelled slides were sequentially scanned as 20 z-sections with an interval of 0.25 µm for all the area of interest (distance from Bregma: −2.92). TH positive (TH+) cells were counted with an automatic protocol with the Volocity 5.4 3D imaging software (PerkinElmer, Coventry, United Kingdom).

### Statistical Analysis

For each data set, the mean (m) and standard deviations (SD) were calculated. A two tailed unpaired Student’s *t-*test was used to evaluate the significance of differences between each data set and the corresponding control. Differences were considered statistically significant when P values were <0.05. For multiple comparisons in *in vivo*, as was the case for comparison. Two-tailed ANOVA followed by a Bonferroni posttest for multiple comparisons was used, with P < 0.05 representing significance.

Wild-type FVB and *Prnp* KO strains mice used in this study were housed at the SISSA mouse facility. Animals handling and subsequent procedures were in accordance with European [European Communities Council Directive of November 24, 1986 (86/609/EEC)] and Italian laws (D.L. 04.03.2014, n°26) and were approved by SISSA Board for Animal Welfare.

### Data and materials availability

All data associated with this study are in the body of the manuscript and listed in the Supplementary Materials.

## Electronic supplementary material

Supplemetary Materials
