# Supplementary material for: α-Synuclein Amyloids Hijack Prion Protein to Gain Cell Entry, Facilitate Cell-to-Cell Spreading and Block Prion Replication
Source: Sci Rep. 2017 Aug 30;7:10050. doi: 10.1038/s41598-017-10236-x (PMC5577263; doi:10.1038/s41598-017-10236-x)
Supplement: Supplementary file 1 — Supplemetary Materials [file 41598_2017_10236_MOESM1_ESM.pdf]

Supplementary Materials for

α-Synuclein Amyloids Hijack Prion Protein to Gain Cell Entry, Facilitate Cell-to-Cell Spreading and Block Prion Replication

Suzana Aulić, Lara Masperone, Joanna Narkiewicz, Elisa Isopi, Edoardo Bistaffa, Elena Ambrosetti, Beatrice Pastore, Elena De Cecco, Denis Scaini, Paola Zago, Fabio Moda, Fabrizio Tagliavini, Giuseppe Legname

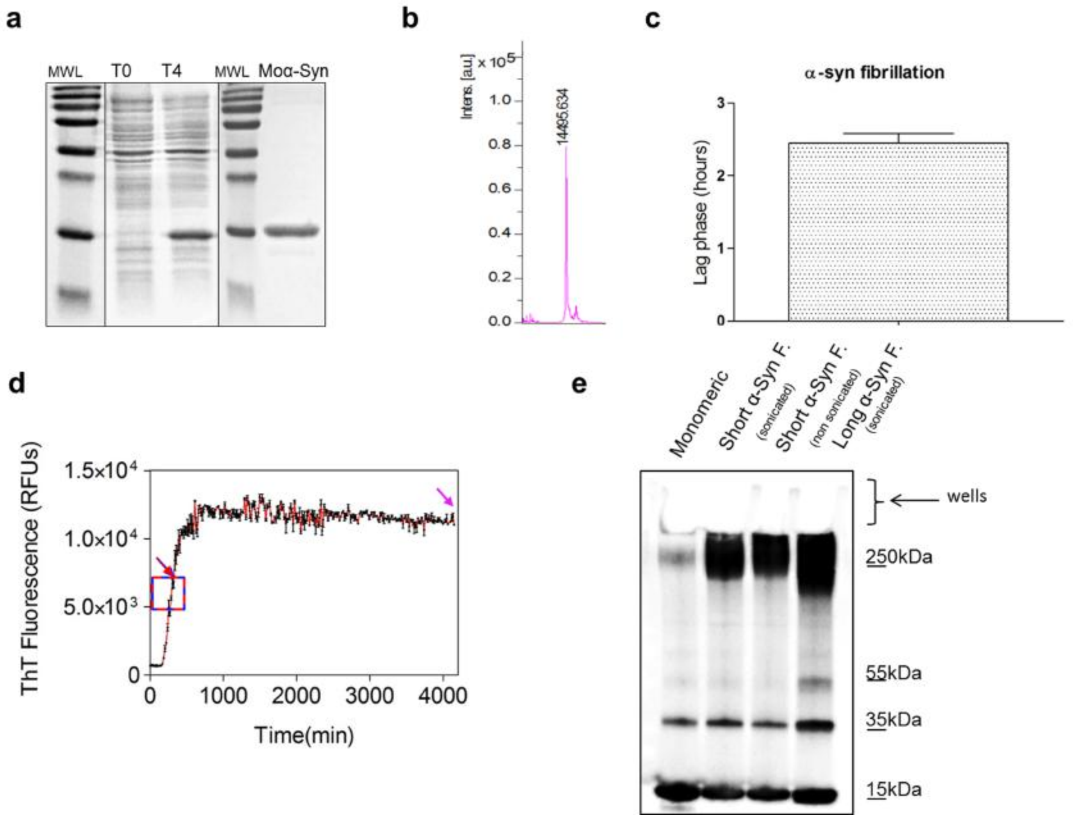

Supplementary Figure S1 | Expression and purification of recombinant α-Syn protein. (**a**) Purification steps of mouse (Mo α-Syn,) α-Syn protein. M: protein molecular weight markers; T0: whole-cell lysate from un-induced cells; T4: whole-cells lysate after 4 hours induction with 0.6 mM IPTG; Mo-Syn: purified α-Syn protein (mouse). (**b**) Mass spectrometry confirmed molecular masss of the protein (Mo α-Syn). (**c**) Lag phase of fibril formation for mouse α-Syn protein. The mouse α-Syn protein forms fibrils fast(2.4 ± 0.23 hours). Data are presented as mean ± SD of three experiments, four replicas for each. (**d**) Fibrillation curve of recombinant mouse α-Syn protein with the time course of ThT changes. The red-blue arrows and square indicates the collection of short α-Syn fibrils, while the pink arrow shows the final time point for the collection of long α-Syn fibrils. (**e**) Western blot depicts the biochemical profile of short amyloid fibrils before and after 5 min sonication, together with long amyloid fibrils that were sonicated (Long α-Syn fibrils sonicated).

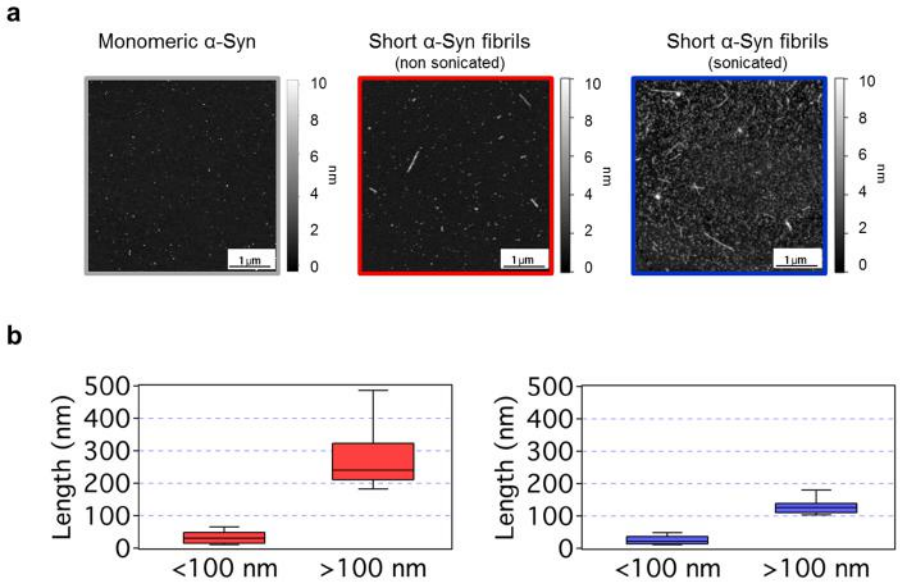

Supplementary Figure S2 | AFM analysis of different α-Syn protein forms. (**a**) AFM images of monomeric and fibrillar α-Syn particles (grey monomeric, red non sonicated, blue sonicated). (**b**) Length quantification of fibrillar α-Syn before and after sonication process (red non sonicated, blue sonicated). Data are represented as mean ± SD.

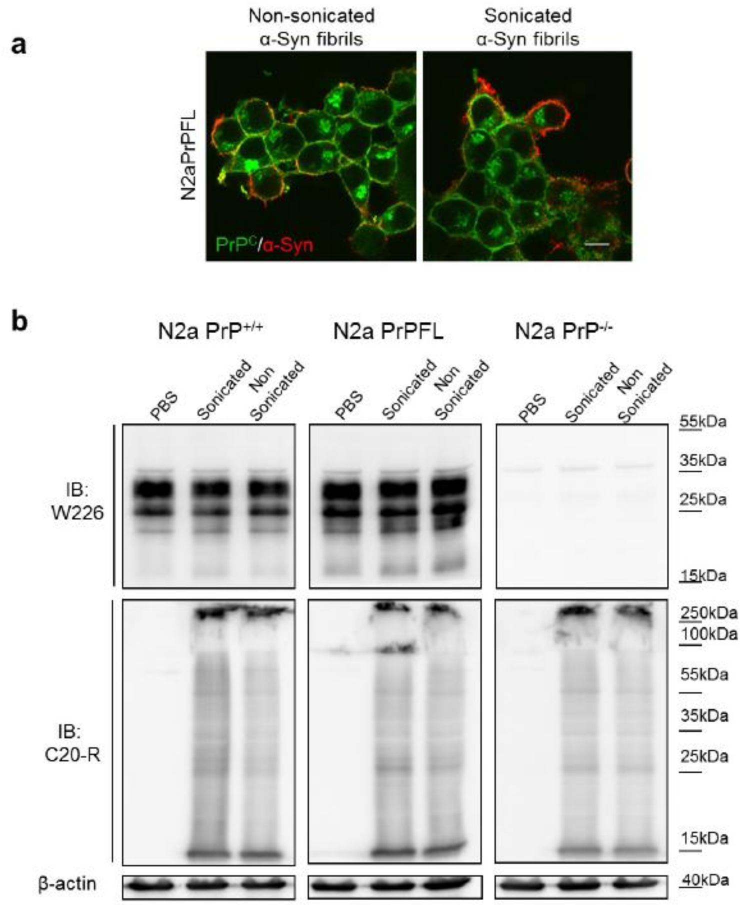

Supplementary Figure S3 | PrP and α-Syn proteins after the incubation with non-sonicated and sonicated mouse α-Syn fibrils. (**a**) Immunofluorescence of N2aPrP$^{-/-}$ cells transfected with full-length PrP (N2aPrPFL, green) shows the co-localization with the exogenously added α-Syn amyloid fibrils (red) on the cell membrane. Scale bar 15 µm. (**b**) Western blots depict PrP^{C} and α-Syn protein levels after treatment with α-Syn amyloid preparations. Each line was loaded with 30 µg of total protein. β-Actin was used as a loading control.

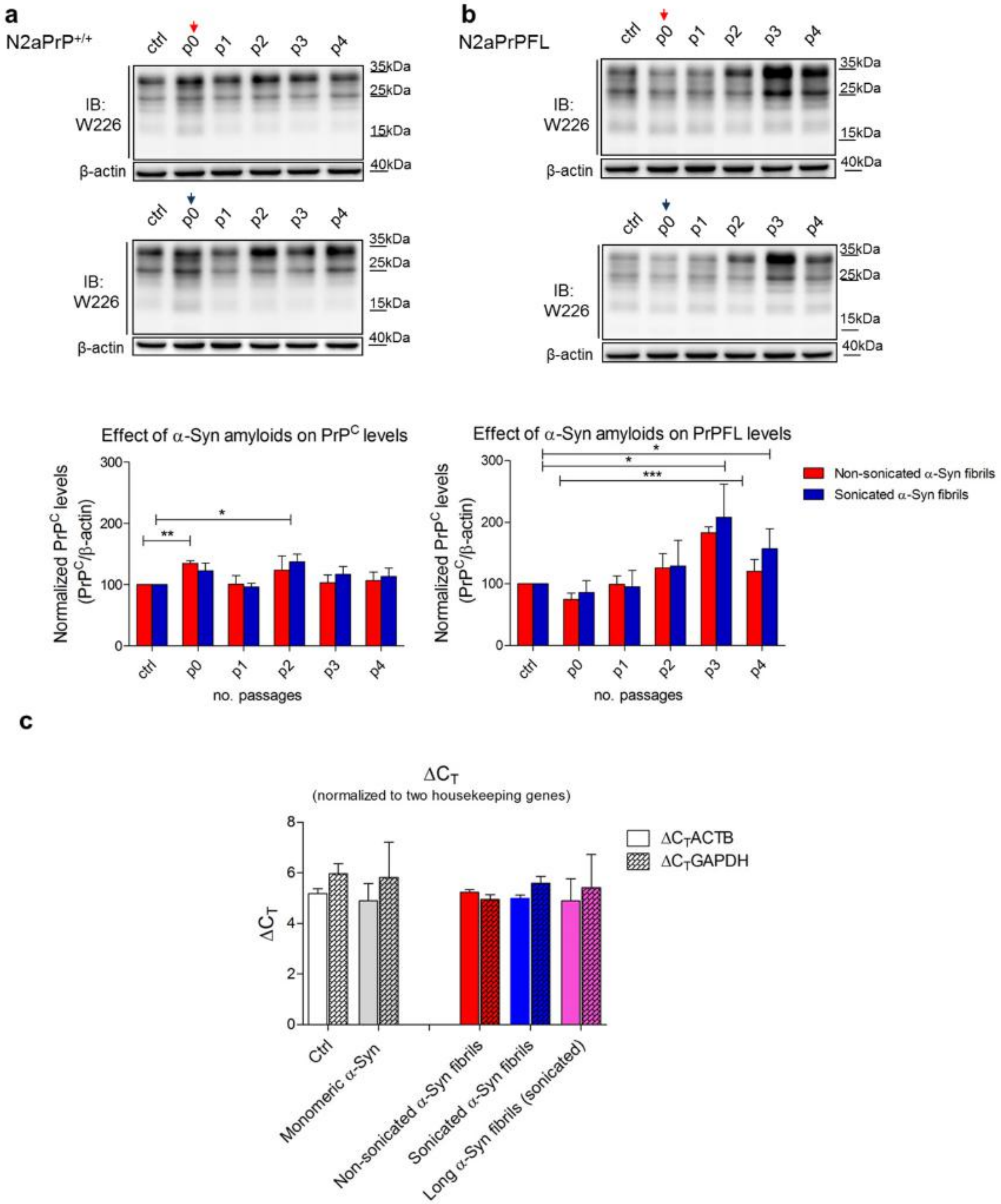

Supplementary Figure S4 | Effect of mouse α-Syn preparations on endogenous PrP^C and transfected PrPFL protein levels in N2a cells. (**a**) The cell lysates of N2a $PrP^{+/+}$ cells and (**b**) transfected N2aPrPFL cells were prepared as described in Materials and Methods and analyzed by SDS-PAGE and WB, using W226 anti-PrP antibody. Red and blue arrows at P0 indicate the treatment with non-sonicated and sonicated α-Syn amyloid fibrils, respectively). Each line was loaded with 30 μg of total protein. Lower graphs show the quantification of three independent experiments, after treatment with non-sonicated α-Syn amyloids (red columns) and sonicated α-Syn amyloids (blue columns). The values are shown as a percentage of total PrP relative to actin. Data are represented as mean ± SD. Data were evaluated by unpaired *t*-test. Statistical analysis is indicated as:

* $P < 0.05$, ** $P < 0.01$, *** $P < 0.001$. (**c**) RT-PCR analysis of N2a non-treated and treated samples. ΔC_T values for *Prnp* gene shows no variability among control and α-Syn treated samples. Normalization of RT-qPCR data was performed on two housekeeping genes, ACTB (empty column) and GAPDH (diagonal brick pattern).

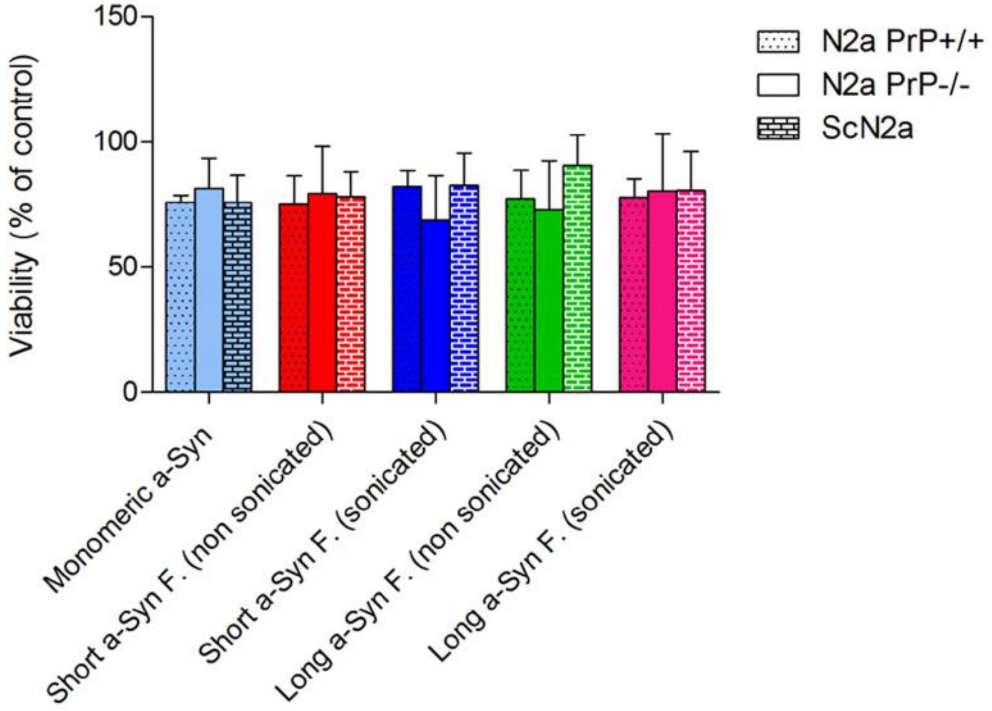

Supplementary Figure S5 | *In Vitro* cell cytotoxicity. Effect of different α-Syn forms (monomeric and fibrils) on growth of N2a $PrP^{+/+}$, N2a $PrP^{-/-}$, ScN2a cells measured by the MTT assay. Data are shown as mean ± SD of three separate experiments, each performed in six replicates.

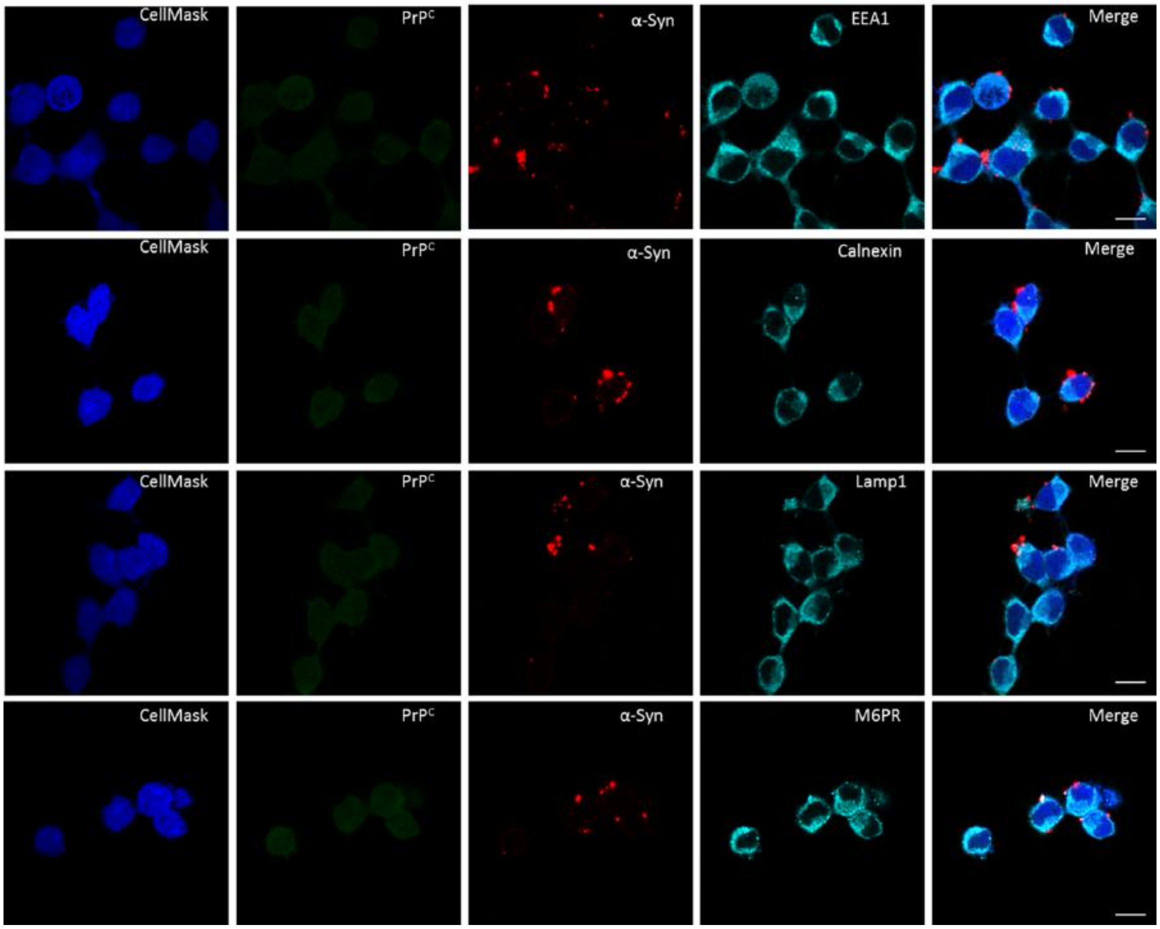

Supplementary Figure S6 | Detection of α-Syn fibrils in cellular compartments by immunofluorescence in N2a PrP$^{-/-}$ cells. Confocal quadruple-labeled immunofluorescent images on N2a PrP$^{-/-}$ cells show the localization of the α-Syn fibrils (red) outside the cells. Other cellular compartments were investigated (EE1, early endosome; Calnexin, endoplasmatic reticulum; LAMP1, lysosomes; Mannose 6 Phosphate Receptor-M6PR, Golgi apparatus). Scale bars 10 μm.

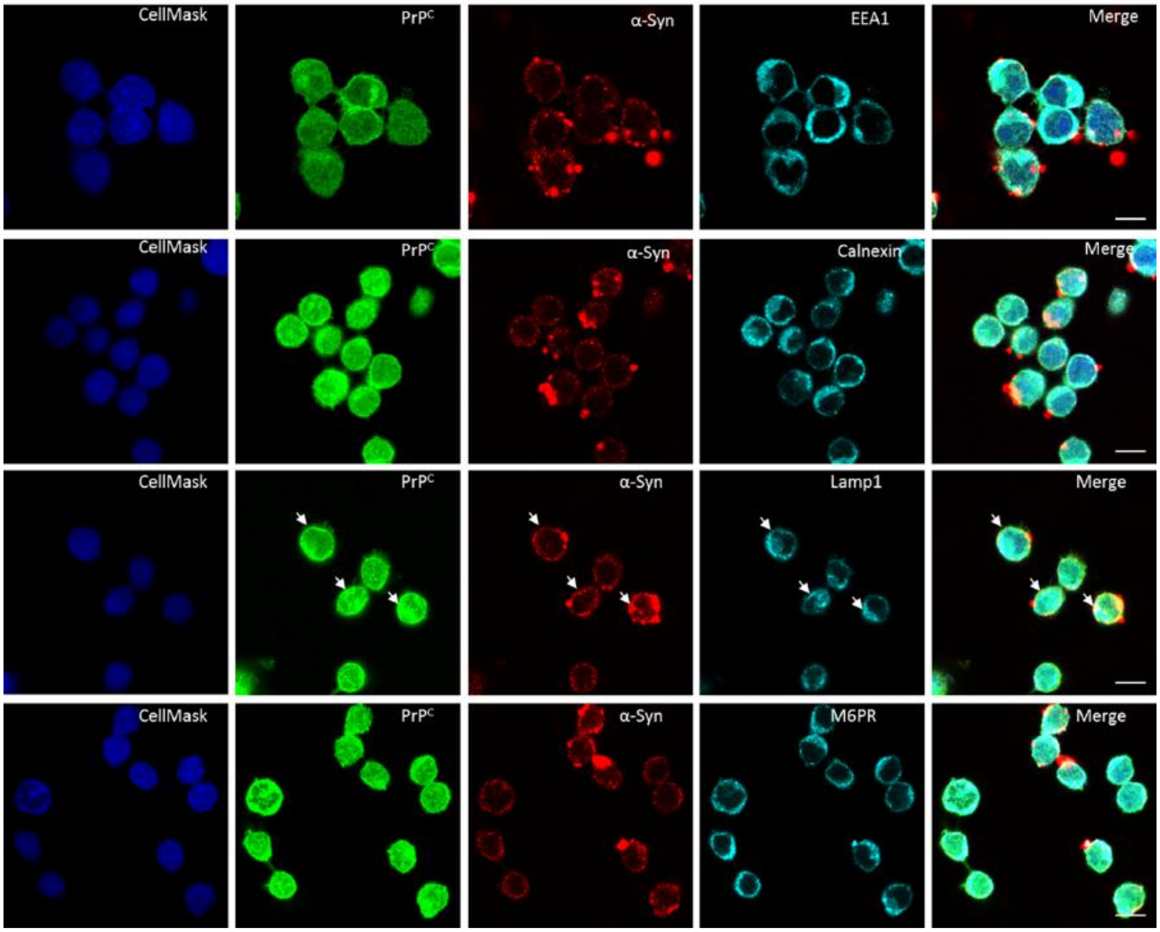

Supplementary Figure S7 | Detection of α-Syn fibrils in cellular compartments by immunofluorescence in N2a PrP$^{+/+}$ cells. Confocal quadruple-labeled immunofluorescent images on N2a PrP$^{+/+}$ cells show the localization of the α-Syn fibrils (red) in the lysosomal compartments (LAMP1, cyan). Other cellular compartments were investigated (EE1, early endosome; Calnexin, endoplasmatic reticulum; Mannose 6 Phosphate Receptor-M6PR, Golgi apparatus). Scale bars 10 µm.

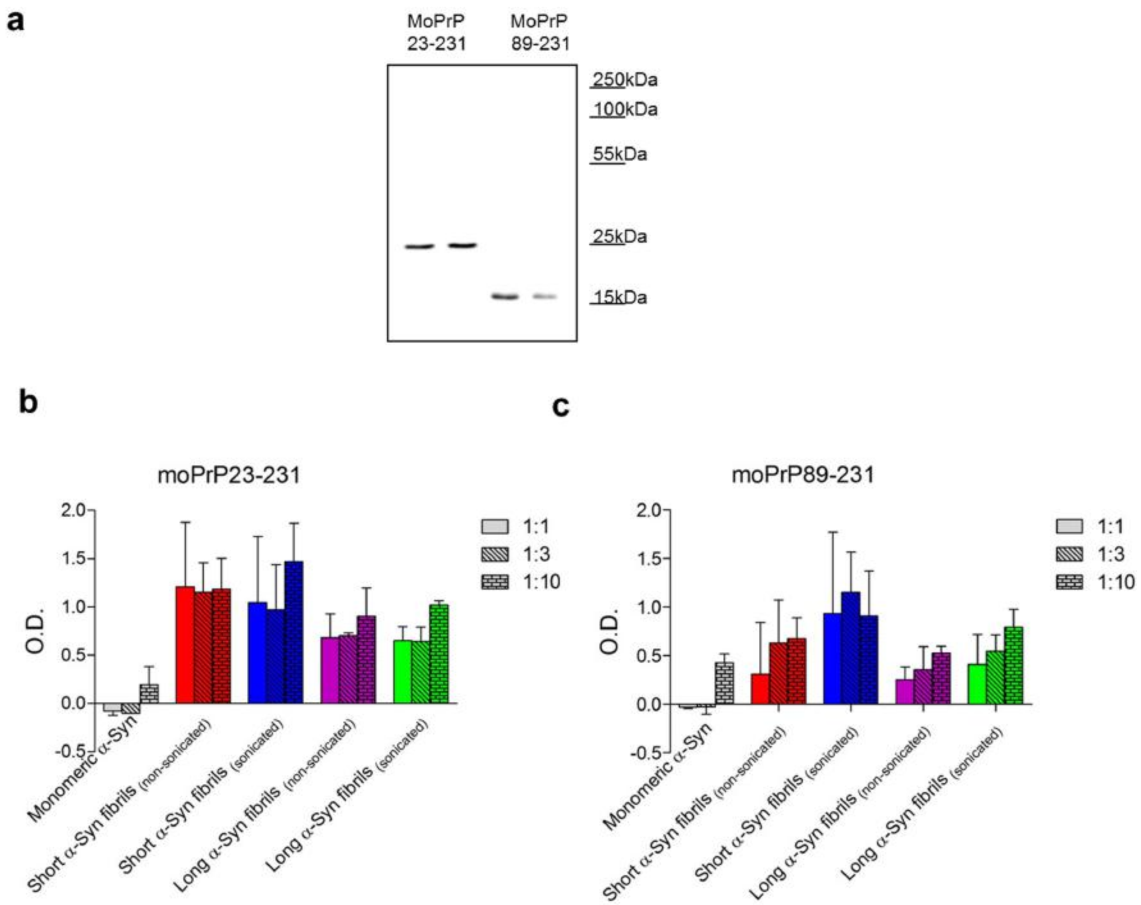

Supplementary Figure S8 | Recombinant PrP protein binds α-Syn amyloids. (**a**) Western blot of recombinant full-length and truncated PrP protein (MoPrP(23-231) and MoPrP(89-231)); (**b** and **c**) ELISA plates were precoated with 50 ng of recPrP and three different ratios of different forms α-Syn (1:1, 1:3, 1:10) were incubated for 30 min at 37 °C. Values are given as mean ± SD.

a

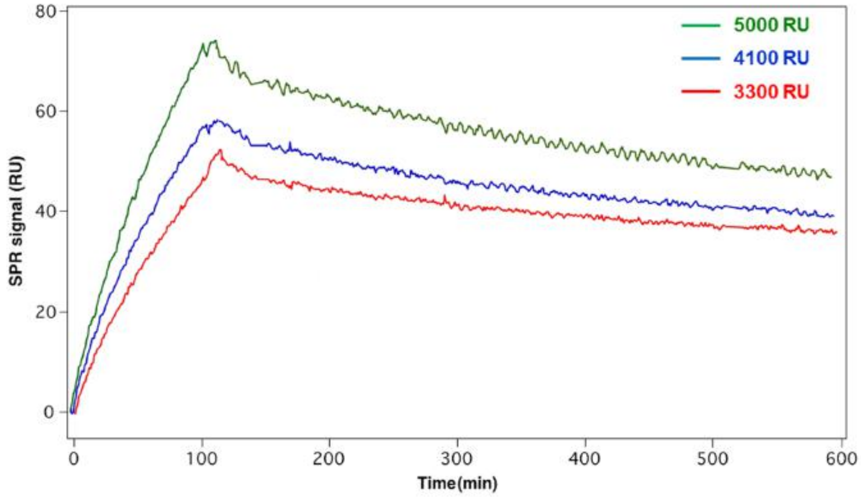

b

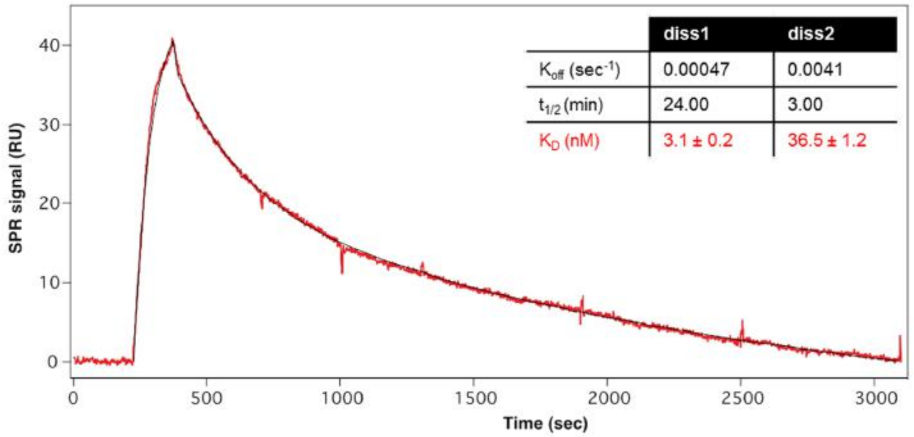

Supplementary Figure S9 | Binding of monomeric and fibrillar α-Syn particles to immobilized sonicated α-Syn fibrils on CM5 biosensor chip. (**a**) Addition of monomeric α-Syn to sonicated α-Syn amyloid surfaces. Three different densities (3300, 4100, and 5000 RU) of sonicated α-Syn amyloid fibrils were immobilized in separate flow cells. Monomeric α-Syn was injected at a concentration of 3 μM over the biosensor chip for 3 min at 50 μL/min association phase) and afterwards flushing with running buffer (dissociation phase). Figure Shows the interaction with monomeric α-Syn as a positive control. (**b**) SPR sensogram shows MoPrP(23-231) binding to α-Syn amyloid fibrils immobilized on CM5 biosensor chip. After the immobilization, soluble recombinant full-length MoPrP (100 nM) was injected across the biosensor chip (binding phase) followed by the injection of buffer alone (dissociation phase). The sensogram showed that the full-length MoPrP binds to the sonicated α-Syn amyloid fibrils. Association and dissociation phases of full-length MoPrP interaction with sonicated α-Syn amyloid fibrils were elaborated using double referencing and analyzed separately: the association phase was fitted with a single exponential equation, determining a unique value of k_{on}; the dissociation phase was analyzed with a double exponential equation, since the sensogram was more adequately fitted by a biphasic model (significantly improved error parameters). The included table indicates the K_D values calculated using the formula $K_D=K_{on}/K_{off}$.

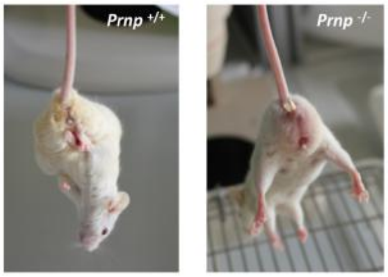

Supplementary Figure S10 | Altered hindlimb clasping behavior in *Prnp*$^{+/+}$ mice and normal behavior in *Prnp*$^{-/-}$ mice. Example of hindlimb clasping: hindlimb clasping behavior towards the abdomen in *Prnp*$^{+/+}$ mice, and hindlimbs splayed outwards away from abdomen in *Prnp*$^{-/-}$ mice like in control mice.

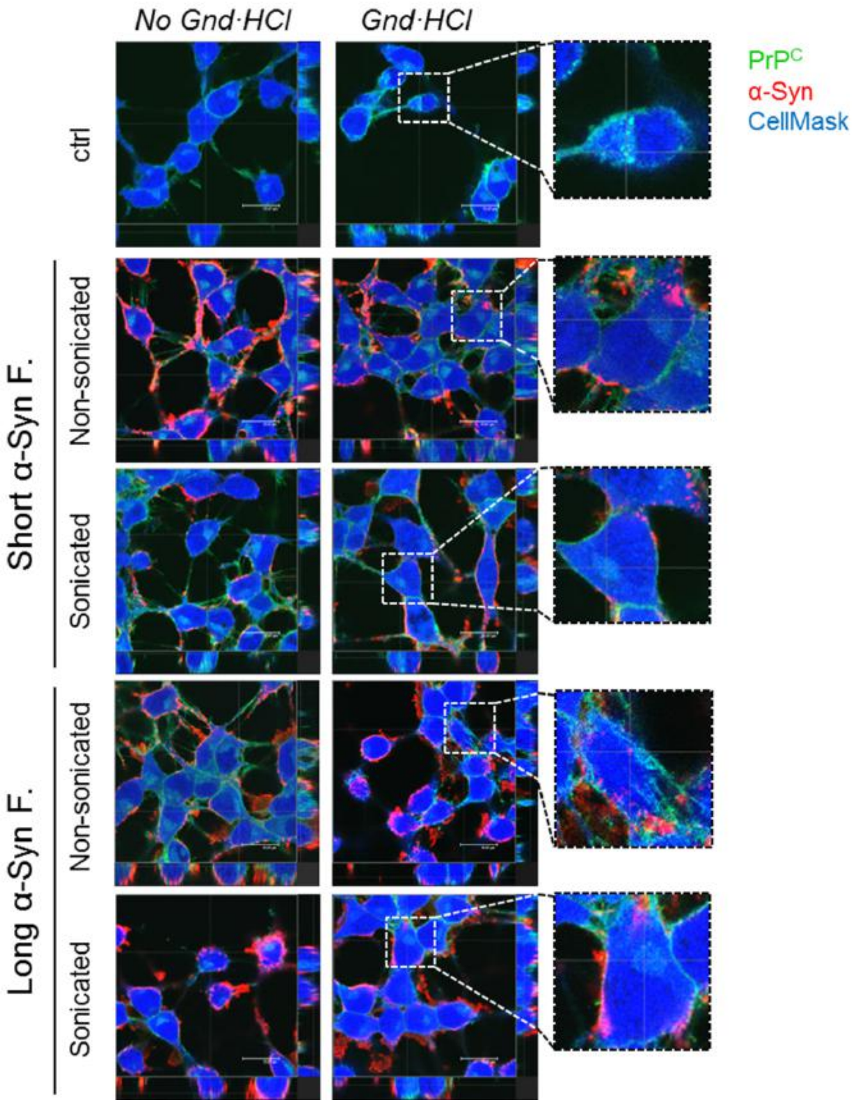

Supplementary Figure S11 | Immunofluorescence (IF) analysis of ScN2a cells treated with α-Syn amyloids show decreased levels of PrP^{Sc}. Denaturation with 6 M GdnHCl permits the detection of PrP^{Sc} in ScN2a cells by IF (ctrl). Cells treated with α-Syn amyloids do not show PrP^{Sc} deposits following the denaturation with 6M GndHCl. Scale bars 15 µm.

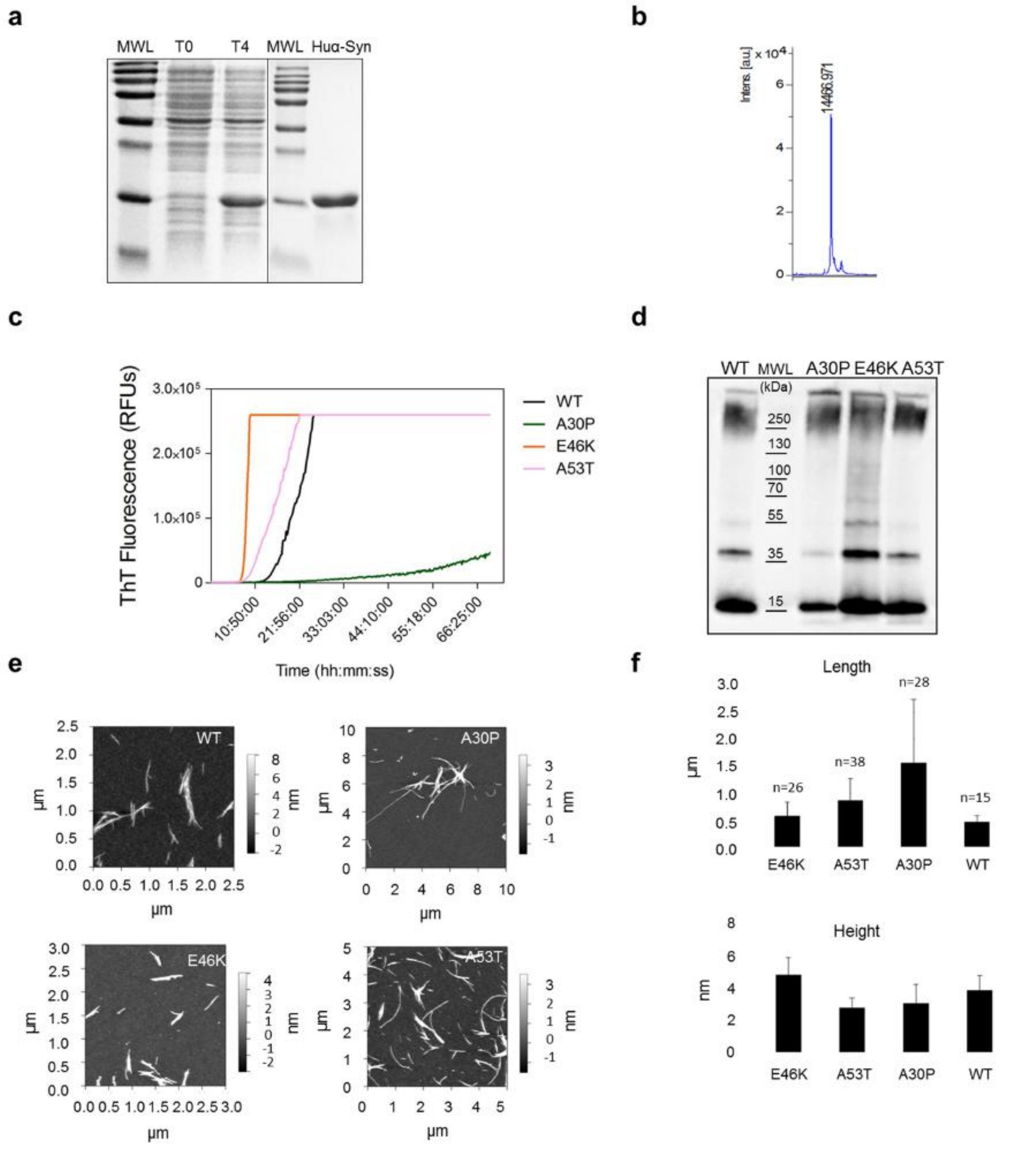

Supplementary Figure S12 | Characterization of human α-Syn fibrils. (**a**) Purification steps of human (Hu α-Syn,) α-Syn protein. M: protein molecular weight markers; T0: whole-cell lysate from un-induced cells; T4: whole-cells lysate after 4 hours induction with 0.6 mM IPTG; Hu-Syn: purified α-Syn protein (human). (**b**) Mass spectrometry confirmed molecular masss of the protein (Hu α-Syn). (**c**) Fibrillation assay of human WT, and three PD-associated mutants (A30P, E46K, and A53T) show different lag phases of fibril formation. (**d**) Western blot of human WT, A30P, E46K, and A53T fibrils shows the migration of all species present in the sample. (**e**) AFM images of human α-

Syn fibrillary species and (**f**) corresponding quantification of length and height. Data are shown as mean ± SD.

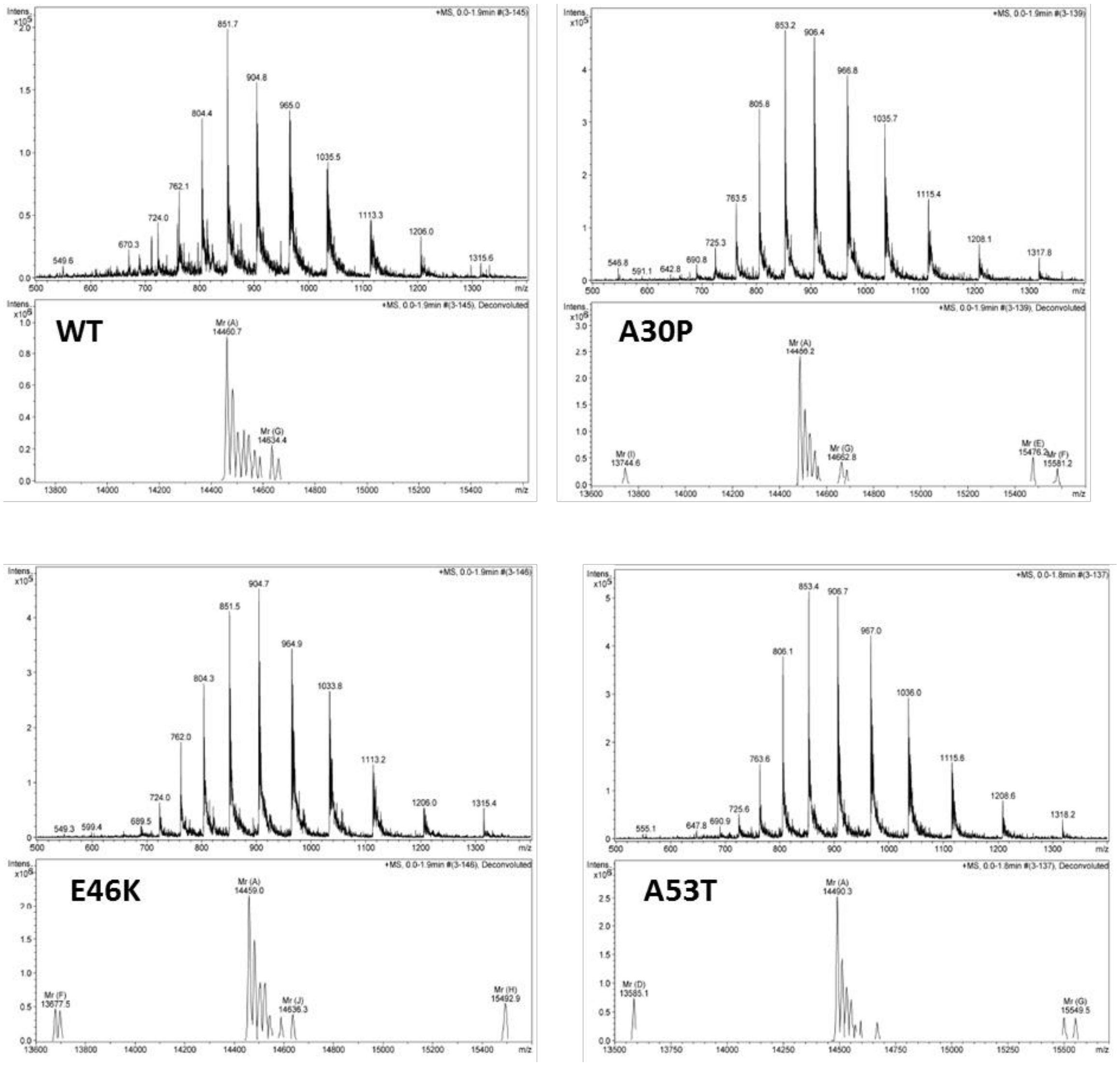

Supplementary Figure S13 | Mass spectroscopy of human WT α-Syn and three PD associated mutants (A30P, E46K and A53T). The molecular weight of WT, A30P, E46K and A53T measured are 14460.7, 14486.2, 14459.0, 14490.3 Da respectively, while those theorical predicted 14460.2, 14486.2, 14459.2, 14490.2 Da

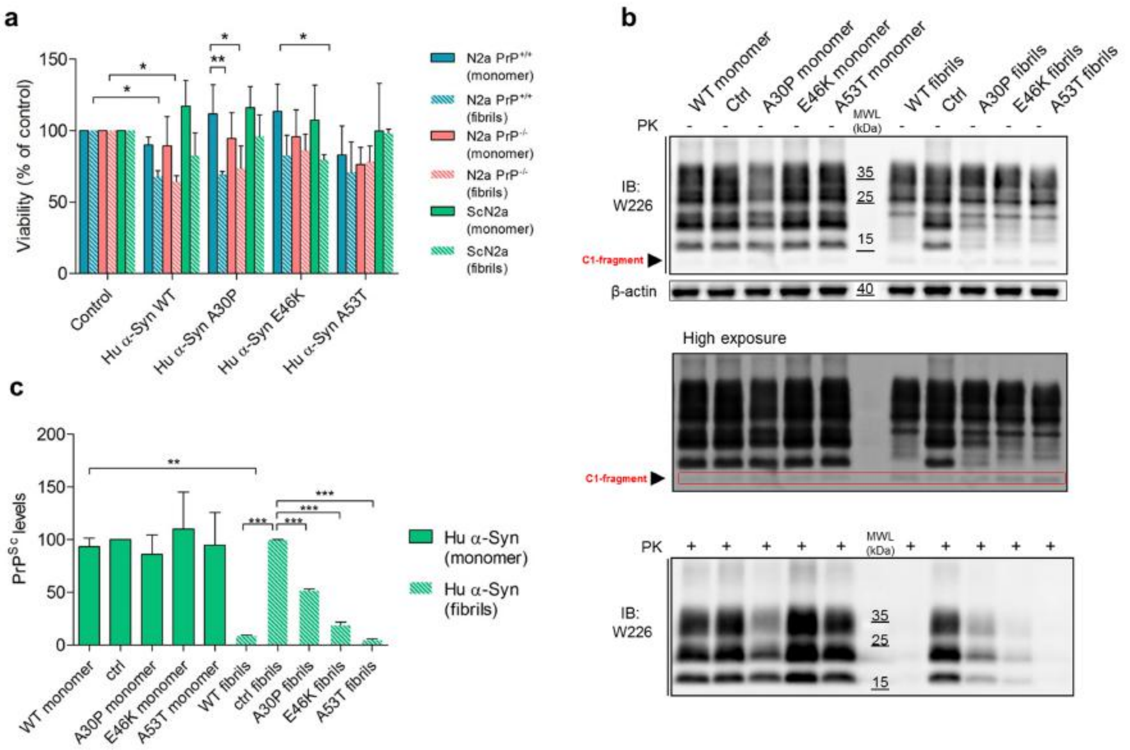

Supplementary Figure S14 | *In vitro* effect of human α-Syn species (monomeric and fibrillary) on cells. (**a**) Effect of different human α-Syn forms (WT, A30P, E46K and A53T, monomers and fibrils) on growth of N2a $PrP^{+/+}$, N2a $PrP^{-/-}$, ScN2a cells measured by the MTT assay. Data are shown as mean±SD of three separate experiments, each performed in six replicates. Statistical significance was determined by using unpaired t-test. Statistical analysis is indicated as: * P < 0.05, ** P < 0.01. (**b**) Immunoblots of ScN2a lysates after treatment for four days with monomeric and fibrillar human α-Syn forms, without (-) and with (+) Proteinase K (PK) digestion. High exposure shows that the treatment with α-Syn fibrils increases the production of C1 fragment. (**c**) Quantification of (**b**), n=3 independent experiments. Values are given as mean ± SD. Statistical significance was determined by using unpaired t-test. Statistical analysis is indicated as: * P < 0.05, ** P < 0.01, *** P < 0.001.

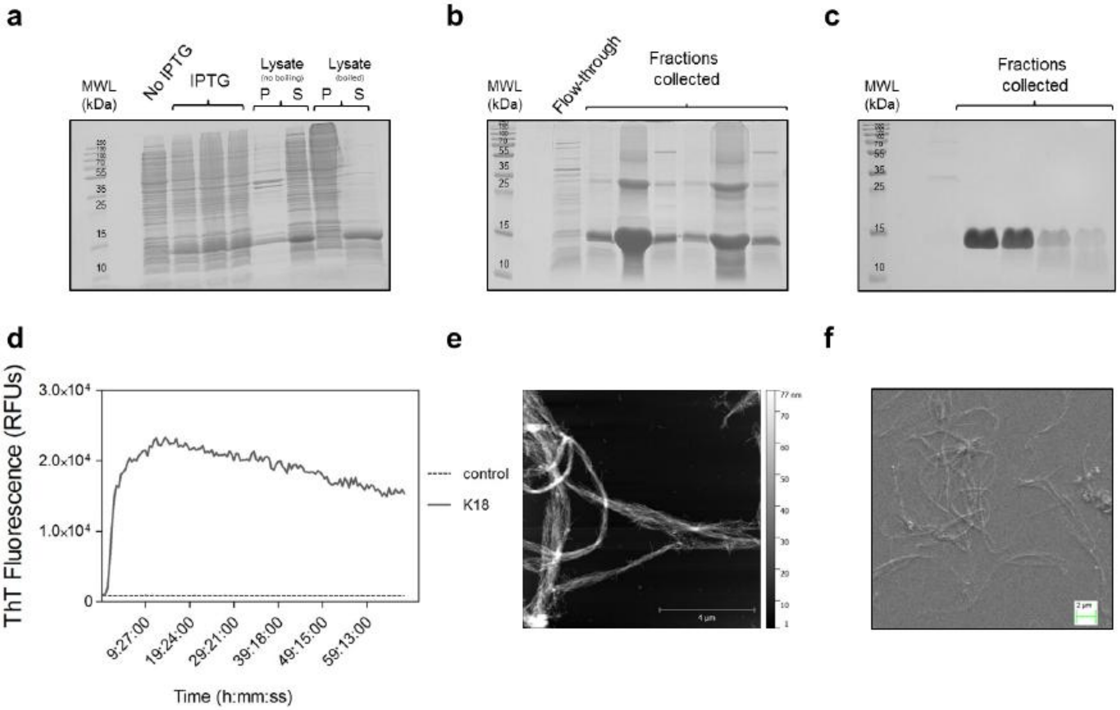

Supplementary Figure S15 | Production and biochemical characterization of tau fragment (K18). (**a**) Purification steps of human tau fragment (K18). MWL: protein molecular weight markers; no-IPTG: whole-cell lysate from un-induced cells; IPTG: whole-cells lysate after induction with 0.5 mM IPTG; Lysate no boiling - Lysates before boiling, P-pellet, S-supernatant; Lysate boiling - Lysates after boiling, P-pellet, S-supernatant; (**b**, and **c**) Cation exchange chromatography fraction collection. (**d**) Fibrillation of tau fragment (fibrillation condition: 0.5 mg/mL tau protein, 50 µg/mL heparin, 0.1 mM DTT in PBS pH 7.4. 10 µM ThT). AFM (**e**) and SEM (**f**) images of fibrillar tau fragment K18.

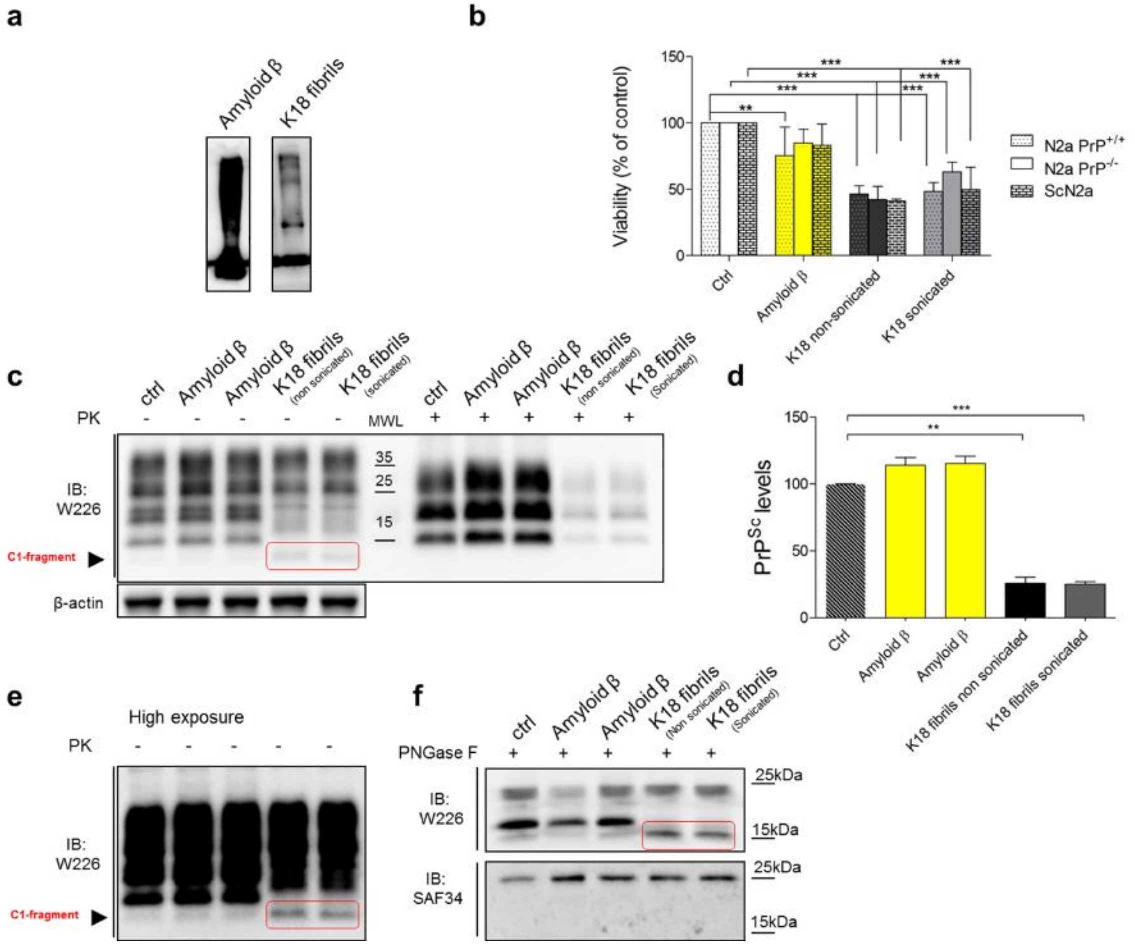

Supplementary Figure S16 | Amyloid-β and tau fragment K18. (**a**) Western blots of amyloid-β and tau fragment K18 in their fibrillary forms show migration of different forms (aggregates, oligomers and monomers). (**b**) Effect of Amyloid-β and tau fragment K18 on growth of N2a $PrP^{+/+}$, N2a $PrP^{-/-}$, ScN2a cells measured by the MTT assay. Data are shown as mean±SD of three separate experiments, each performed in six replicates. Statistical significance was determined by using unpaired t-test. Statistical analysis is indicated as: * P < 0.05, ** P < 0.01, *** P < 0.001. (**c**) Immunoblots of ScN2a lysates after treatment for four days with Amyloid-β and tau fragment K18 fibrils, without (-) and with (+) Proteinase K (PK) digestion. (**d**) Quantification of (**c**), n=3 independent experiments. Values are given as mean ± SD. Statistical significance was determined by using unpaired t-test. Statistical analysis is indicated as: * P < 0.05, ** P < 0.01, *** P < 0.001. (**e**) High exposure of the Western blot shown in (**c**) (non-digested with PK(-)), shows that the treatment with K18 fibrils increases the production of C1 fragment. (**f**) C1 production in ScN2a cells was confirmed after PNGase F digestion when the membranes were probed with two antibodies (W226 is a C-terminal Ab, SAF34 is N-terminal Ab).

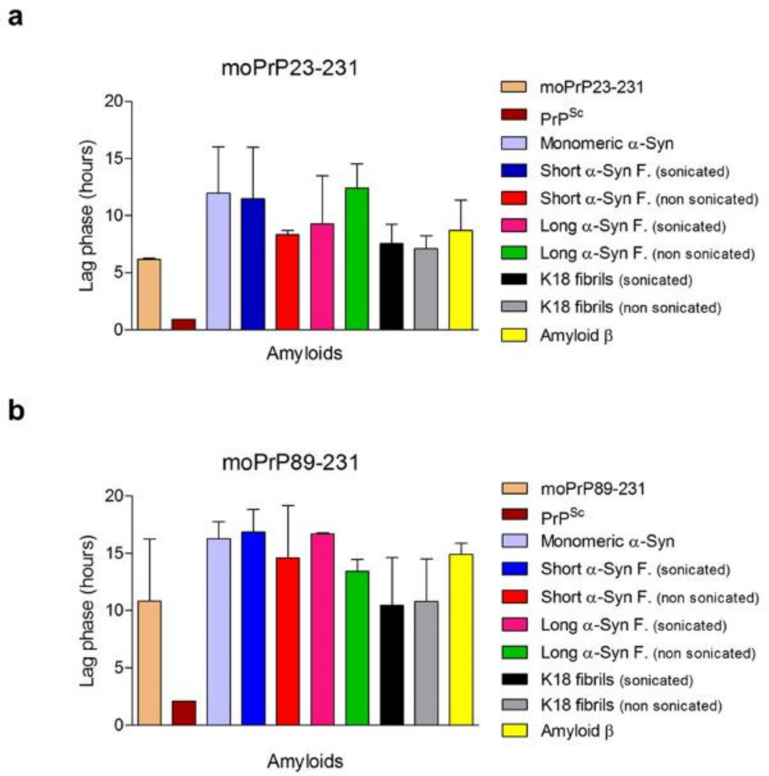

Supplementary Figure S17 | Analysis of the length of the lag-phase of amyloid seeding assay curves of recPrP protein in the presence of various amyloid seeds. (**a**) The effect of adding various amyloids in the presence of full-length moPrP23-231 protein: the addition of PrP^{Sc} seed confirmed the shortening of the lag phase; Amyloid β, K18 tau fragment (non sonicated and sonicated fibrils), and monomeric α-Syn did not had any effect of the process of fibril formation of moPrP23-231 protein (same lag phase as control condition); all fibrillar forms of mouse α-Syn delayed the lag phase of fibril formation of the full-length moPrP23-231 protein. Data are reported as the mean ± SD of triplicate experiments. (**b**) The effect of adding various amyloids in the presence of truncated moPrP89-231 protein: the addition of PrPSc seed confirmed the shortening of the lag phase; K18 tau fragment (non sonicated and sonicated fibrils) did not had any effect of the process of fibril formation of moPrP89-231 protein (same lag phase as control condition); all fibrillar forms of mouse α-Syn, amyloid β, and monomeric α-Syn delayed the lag phase of fibril formation of the full-length moPrP89-231 protein. Data are reported as the mean ± SD of triplicate experiments.
